# GC content mismatch of transgene destabilizes RNA virus genomes

**DOI:** 10.1128/jvi.00882-26

**Published:** 2026-07-13

**Authors:** Sachiho Kawahara, Naganori Nao, Sarun Tulakarnwong, Saori Suzuki, Rigel Suzuki, Sarin Chimnaronk, Tomokazu Tamura, Takasuke Fukuhara

**Affiliations:** 1 School of Medicine, Hokkaido University 12810, Sapporo, Japan; 2 Department of Microbiology and Immunology, Faculty of Medicine, Hokkaido University 12810, Sapporo, Japan; 3 One Health Research Center, Hokkaido University 12810, Sapporo, Japan; 4 Institute for Vaccine Research and Development (HU-IVReD), Hokkaido University 12810, Sapporo, Japan; 5 Division of International Research Promotion, International Institute for Zoonosis Control, Hokkaido University 12810, Sapporo, Japan; 6 Institute of Molecular Biosciences, Mahidol University 26685, Salaya, Nakhon Pathom, Thailand; 7 Department of Virology, Faculty of Medical Sciences, Kyushu University 12923, Fukuoka, Japan; 8 Siriraj Center of Research Excellence for Systems Pharmacology, Department of Pharmacology, Faculty of Medicine Siriraj Hospital, Mahidol University 26685, Bangkok, Thailand; 9 Institute for Genetic Medicine, Hokkaido University 12810, Sapporo, Japan; 10 Laboratory of Virus Control, Research Institute for Microbial Diseases, The University of Osaka 13013, Suita, Japan; 11 AMED-CREST, Japan Agency for Medical Research and Development (AMED), Tokyo, Japan; St Jude Children's Research Hospital, Memphis, Tennessee, USA

**Keywords:** RNA viruses, GC content, RNA structure, codon usage, reporters

## Abstract

GC content—the proportion of guanine and cytosine nucleotides—varies widely among organisms and viruses. Although GC content is recognized to have biological significance, its functional role in viruses remains poorly understood. Here, we examined the impact of GC-content bias using three positive-sense single-stranded RNA viruses with distinct GC profiles: severe acute respiratory syndrome coronavirus-2 (SARS-CoV-2), Japanese encephalitis virus (JEV), and hepatitis C virus (HCV). Variants of the NanoLuc (Nluc) reporter gene, engineered with synonymous mutations to alter GC content, were inserted into each viral genome. Serial passaging experiments revealed strong effects on genome stability and viral fitness. In SARS-CoV-2, which has low GC content, the introduction of a GC-rich Nluc gene disrupted genome stability, leading to frequent deletions of Nluc. In both SARS-CoV-2 and HCV, Nluc with mismatched GC levels accumulated substitutions that optimized GC content toward that of the viral genome, predominantly at the third codon position. tRNA-seq analysis revealed a shift in the host tRNA pool toward a more GC-rich composition during HCV infection, consistent with this substitution bias. In contrast, JEV (intermediate GC content) maintained the Nluc variants across serial passages, suggesting reduced selective pressure. Taken together, these findings demonstrate that mismatches between viral genome GC content and inserted sequences profoundly affect genetic stability, suggesting evolutionary constraints that may shape RNA virus composition more broadly. This study provides mechanistic insight into how GC content influences viral genome maintenance and offers a framework for designing genetically stable recombinant reporter viruses.

**IMPORTANCE:**

GC content is a fundamental genomic feature that influences gene expression, genome architecture, and adaptation. Although its role in cellular genomes has been extensively studied, the functional significance of GC content in viral genomes remains poorly understood. In this study, we show that incompatibility in GC content between a viral genome and an inserted transgene destabilizes RNA secondary structures, compromising viral genome integrity, and is accompanied by changes in the host tRNA pool consistent with this compositional bias. These findings identify GC content as a previously underappreciated determinant of virus–host compatibility and viral fitness. Importantly, our results provide mechanistic insight into how the GC content of a viral genome can be matched to its host cellular environment. By demonstrating that GC content matching is critical for the stability of recombinant viruses, this study provides a basis for the rational design of reporter viruses, vaccine platforms, and antiviral screening tools.

## INTRODUCTION

The guanine (G) and cytosine (C) nucleotide content of a genome—known as the GC content—is a fundamental feature that influences a wide range of biological characteristics. Indeed, GC content differs across species. Humans (*Homo sapiens*) and mice (*Mus musculus*) have approximately 40% GC content in their genomes (1). The platypus (*Ornithorhynchus anatinus*) genome has 45.5% GC content, while the gray short-tailed opossum (*Monodelphis domestica*) has about 38% (2). Interestingly, the genome GC content of microorganisms and other less complex organisms is more variable, ranging from ~13% in *Zinderia insecticola* (3) to as high as 75% in *Aneromyxobacter dehalogenan*s (4). Previous studies have shown that GC content is involved in bacterial survival strategies (5). Furthermore, GC content is known to play a significant role in epigenetics, where it is associated with DNA methylation, transcriptional regulation, and RNA secondary structure formation (6, 7).

Among viral genomes, GC content varies substantially, with certain species exhibiting distinct compositional biases. This diversity is evident in both RNA and DNA viruses. For example, the RNA viruses hepatitis A virus and severe acute respiratory syndrome coronavirus 2 (SARS-CoV-2) are characterized by low GC content (37% and 38%, respectively), whereas rubella virus displays a markedly high GC content of 70%. Similarly, the DNA virus family *Herpesviridae* has substantial compositional diversity, with genome GC content from 32% to 75% (8). However, the functional significance of GC content in viruses has remained poorly understood.

We previously engineered a codon-optimized reporter gene with a GC ratio similar to that of SARS-CoV-2 and showed that it replicated robustly in cell cultures, with kinetics comparable to those of the parental SARS-CoV-2 genome (9). In contrast, the recombinant SARS-CoV-2 carrying reporter genes with mismatched GC content did not produce detectable cytopathic effects (CPEs), suggesting an absence of viral propagation. These findings suggest that the GC content of inserted foreign genes may significantly impact viral replication and fitness. However, it remained unclear how GC mismatch affects genome stability and sequence evolution once a recombinant virus is established, and whether such effects are shared across RNA viruses with different GC profiles.

In the present study, we aimed to elucidate the significance of transgene GC content in viral propagation. To this end, we generated multiple versions of the NanoLuc (Nluc) reporter gene, each carrying synonymous substitutions to alter GC content. We then incorporated these GC-modified Nluc genes into three viruses with varying GC content—SARS-CoV-2 (GC content = 38%), Japanese encephalitis virus (JEV; GC content = 51%), and hepatitis C virus (HCV; GC content = 58%)—and assessed the characteristics of the resultant recombinant genomes. We observed distinct virus-specific outcomes, suggesting that the influence of reporter GC content may depend on the genomic context of the recipient virus.

## RESULTS

### Characterization of GC-modified Nluc

**Fig 1:**
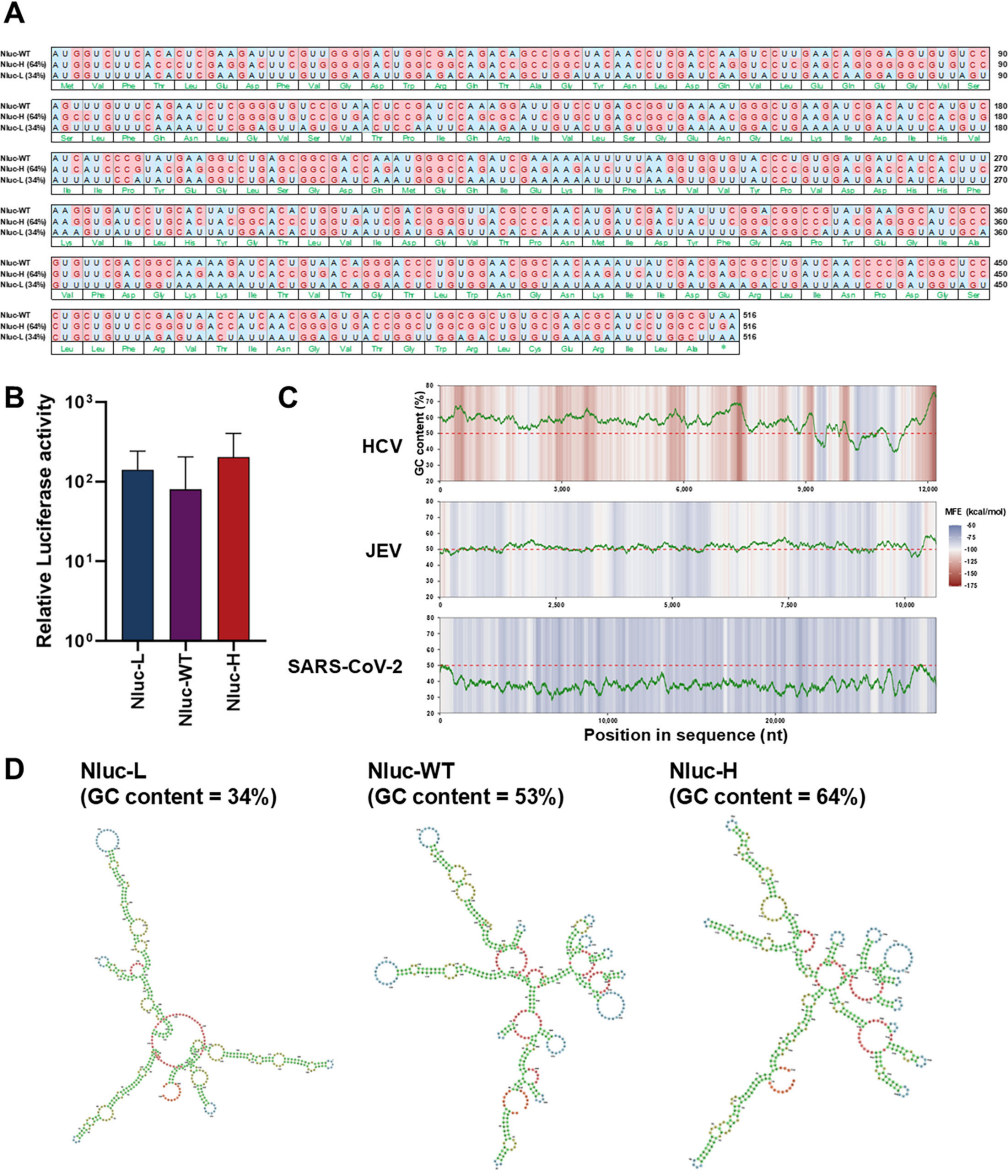
Characterization of three GC-modified NanoLuc (Nluc) variants. (**A**) Nucleotide alignment of wild-type (Nluc-WT; GC content=53%) and GC-modified (GC content = 34% for Nluc-L; 64% for Nluc-H) Nluc. (**B**) The luciferase activity of Nluc-WT, Nluc-L, and Nluc-H expressed under the HSV-TK promoter was evaluated after transfection into HEK293T cells. At 24 h post-transfection, cells were lysed, and Nluc and firefly luciferase activities were evaluated using a luminometer. Firefly luciferase served as a transfection/internal control, and the bar graphs show the normalized data (Nluc/firefly luciferase activities) (*n* = 3). (**C**) The GC content and minimum free energy (MFE) were calculated in 300 nt windows for each parental virus. (**D**) RNA secondary-structure predictions for Nluc with the different GC contents.

To investigate the impact of transgene GC content on viral propagation, we synthesized two mutant Nluc genes carrying synonymous substitutions to alter the GC content (Fig. 1A). Wild-type Nluc (Nluc-WT) has 53% GC content, and we made two mutants with 34% (Nluc-L) or 64% (Nluc-H) GC content.

First, to investigate whether the GC-modified Nluc gene influences translational efficiency, each Nluc was expressed in a vector under the herpes simplex virus thymidine kinase (HSV-TK) promoter. No significant difference in luciferase activities was observed for the three Nluc constructs, indicating that the GC content of Nluc modified in this context did not affect translation efficiency in mammalian cells (Fig. 1B).

### Structural characterization of GC-modified Nluc variants and the three target viruses

Because GC content is known to influence the formation and stability of RNA secondary structures (10, 11), we next examined the predicted structural properties of both the target viruses and the GC-modified Nluc variants to establish a structural framework for the subsequent functional analyses.

We first focused on the viruses, which span a broad range of genome-wide GC content: SARS-CoV-2 (29,895 nucleotides; GC content = 38%) (12), the JFH-1 strain of HCV (9,678 nucleotides; GC content = 58%) (13), and the AT31 strain of JEV (10,926 nucleotides; GC content = 51%) (14). To assess the relationship between local GC content and structural stability, we calculated the GC content and the minimum free energy (MFE) of each viral genome using a 300 nt sliding window with the ViennaRNA package (15) (Fig. 1C). MFE represents the Gibbs free energy of the most thermodynamically stable predicted RNA secondary structure and is used as an index of structural stability (16). The average MFE was −76.40 kcal mol⁻¹ for SARS-CoV-2, −112.51 kcal mol⁻¹ for HCV, and −91.01 kcal mol⁻¹ for JEV. Because more negative MFE values correspond to greater thermodynamic stability of the predicted secondary structure, these results indicate that HCV possesses the most structurally stable genome and SARS-CoV-2 the least, with JEV at an intermediate level. The trend was consistent with the GC content of each genome and is in line with previous reports linking higher GC content to greater RNA structural stability.

We then predicted the RNA secondary structures of the three GC-modified Nluc variants using MXfold2 (17) (Fig. 1D). The MFE values for the full-length RNAs were −119.1 kcal mol⁻¹ for Nluc-L, −173.0 kcal mol⁻¹ for Nluc-WT, and −221.0 kcal mol⁻¹ for Nluc-H, demonstrating that synonymous substitutions alone substantially altered predicted structural stability. To compare the variants more systematically, we calculated a set of structural complexity metrics: base-pair count, helix number, longest helix, hairpin loops, internal loops, multiloop junctions, branching points, and maximum span (Table 1). Across these metrics, the values increased monotonically with GC content, indicating that higher GC content was associated with greater structural complexity in addition to greater thermodynamic stability.

**TABLE 1:** Complexity metrics of RNA structures of the GC-modified Nluc variants

Nluc	Pairs	Stems	MaxStem	Hairpin	Internal	Multiloop	Branching	MaxSpan
Nluc-L (GC content = 34%)	2	1	2	1	0	0	0	7
Nluc-mL (GC content = 46%)	34	16	3	11	2	16	15	466
Nluc-WT (GC content = 53%)	65	27	3	13	10	20	26	444
Nluc-H (GC content = 64%)	93	35	3	17	15	22	34	510

Taken together, these analyses establish that (i) the three viral genomes selected for this study differ markedly in both GC content and predicted RNA structural stability, and (ii) synonymous GC modification of Nluc produces variants with substantially different structural properties despite encoding the same protein and showing comparable basal expression in mammalian cells (Fig. 1B). This set of GC-modified Nluc variants therefore provides a controlled experimental system in which GC content and predicted RNA structure can be modulated independently of basal reporter activity, allowing us to dissect the contribution of transgene GC composition to viral genome behavior in the experiments that follow.

### Generation and characterization of transgenes with varying GC content in the virus with moderate GC content

**Fig 2:**
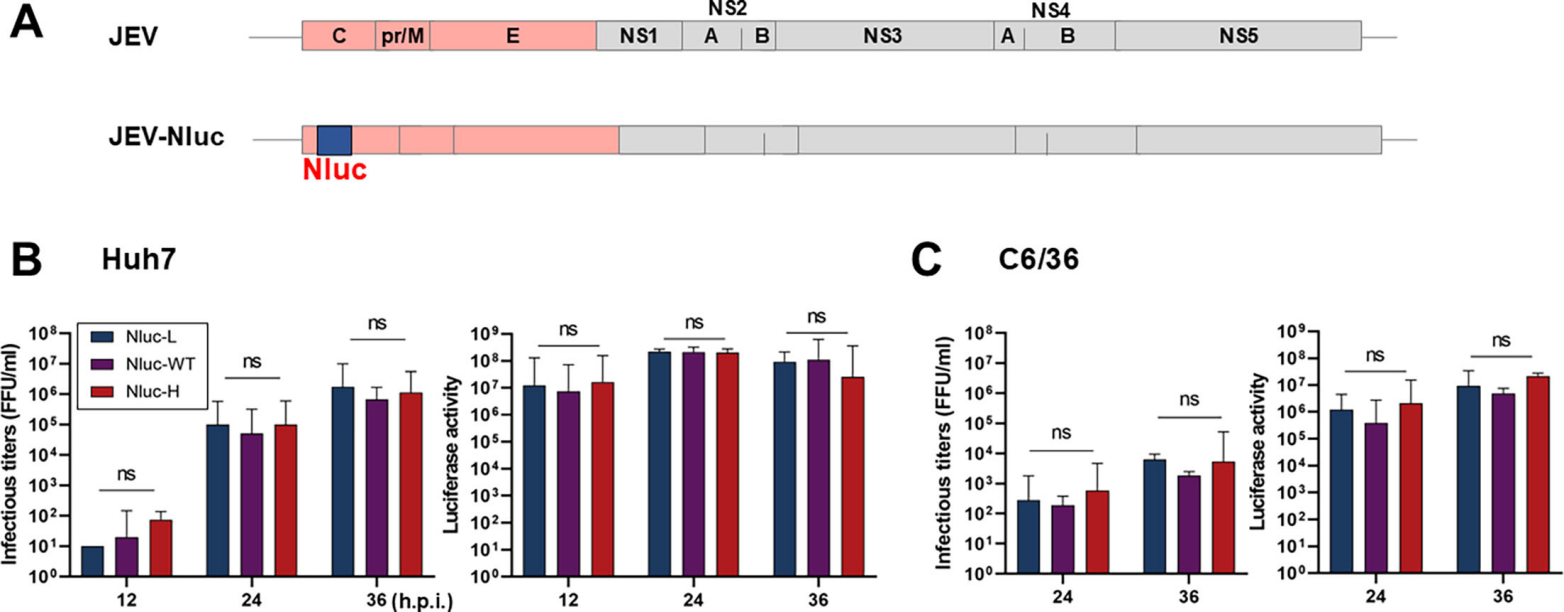
Generation and characterization of the GC-modified Nluc in JEV. (**A**) A schematic representation of the wild-type (WT) JEV and the recombinant JEV carrying the Nluc gene. (**B**) *In vitro* growth kinetics of JEV-Nluc-WT, -L, and -H. Huh7 cells were infected with each of the JEV-Nluc variants (MOI = 0.01). Infectious titers were assessed in the culture supernatants (left panel), and Nluc luciferase activity in the cells (right panel) (*n* = 2). (**C**) *In vitro* growth kinetics of JEV-Nluc-WT, -L, and -H. C6/36 cells were infected with each of the JEV-Nluc variants (MOI = 0.001). Infectious titers were assessed in the culture supernatants (left panel), and Nluc luciferase activity in the cells (right panel) (*n* = 2).

We first investigated JEV, which possesses an intermediate GC content and intermediate predicted genome stability among the three viruses examined here (Fig. 1C). The AT31 strain of JEV (14) was used throughout. We evaluated recombinant JEV carrying the GC-modified Nluc constructs (Fig. 2A). The human hepatocellular carcinoma-derived Huh7 cells were infected with either JEV-Nluc-WT, JEV-Nluc-L, or JEV-Nluc-H, and the replication kinetics of the viruses were evaluated. The infectious titers of culture supernatants (Fig. 2B, left panel) and intracellular luciferase activities (Fig. 2B, right panel) were comparable over time for these recombinant reporter viruses. To further investigate the growth properties of these recombinant JEV-Nluc variants in cell lines derived from other species, the mosquito-derived C6/36 cells were also infected with the respective reporter JEVs. No significant differences in infectious titers (Fig. 2C, left panel) and luciferase activities (Fig. 2C, right panel) were observed among them even in C6/36 cells.

**Fig 3:**
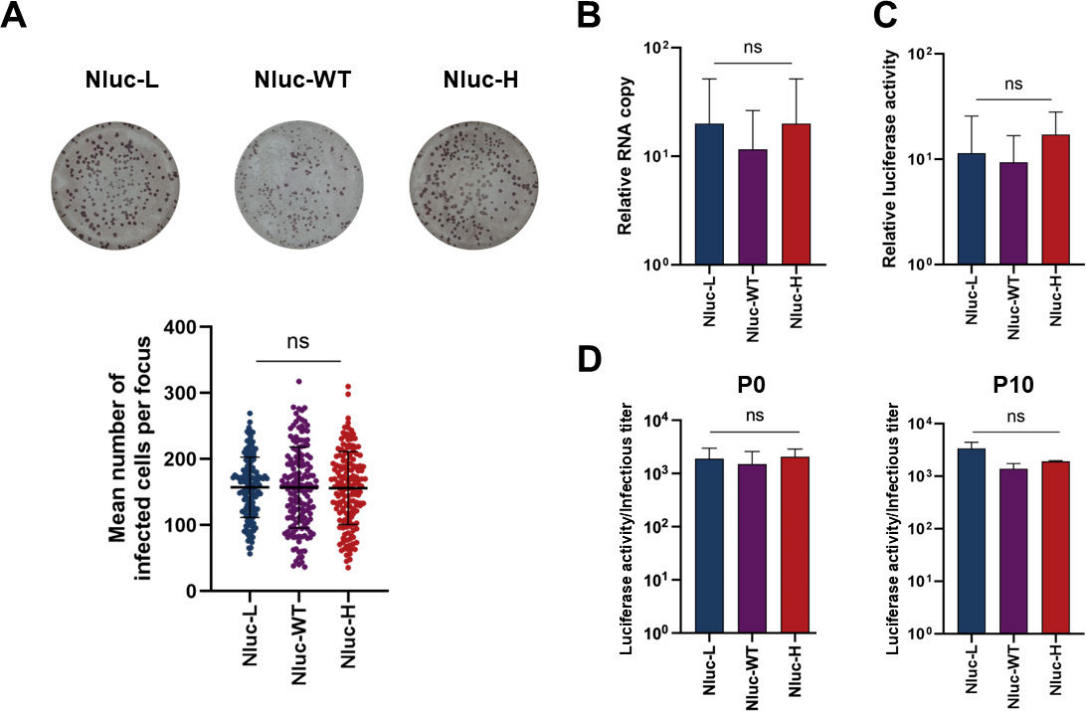
Effects of GC-modified Nluc on replication and genetic stability in JEV. (**A**) The infected cell numbers per FFU were calculated at 48 hpi of JEV-Nluc-WT, -H, and -L. The area of each FFU (upper images are representative for the analysis) was measured for all analyzed FFUs (>100 FFUs per condition) and shown as scatter plots with the mean. (**B**) Viral RNA copy numbers of JEV-Nluc-WT, -L, and -H at 12 h were normalized to the corresponding values at 9 h (*n* = 3). (**C**) Luciferase activity of JEV-Nluc-WT, -L, and -H at 12 h was normalized to the corresponding values at 9 h (*n* = 3). (**D**) Ratio of luciferase activity to infectious titer for each of the recombinant JEVs at passage 0 (P0) vs passage 10 (P10) at 24 hpi (*n* = 2).

Insertion of transgene within the viral genome may affect multiple steps of viral life cycle, including viral entry/spreading, viral gene replication, and viral gene translation. Focus-forming assays were performed for evaluation of the viral entry/spreading. The number of cells per focus-forming unit (FFU) was quantified at 48 hpi (hours post-infection). No significant differences in the number of cells in each focus were observed (Fig. 3A), indicating that the three recombinant viruses exhibited comparable viral spreading ability.

Next, to evaluate the effects of the GC modification of Nluc on viral gene replication and translation, viral intracellular RNA copies and luciferase activities were measured at 9 and 12 hpi for viral gene replication and translation, respectively. As shown in Fig. 3B, no significant differences in the replication kinetics were observed among the three Nluc variants, suggesting these viruses possess comparable viral RNA replication capacities. Similarly, the translational kinetics measured by luciferase intensity were comparable among the viruses (Fig. 3C), indicating that differences in GC content of Nluc had no detectable effect on translational efficiency. Taken together, these findings suggest that the GC-modified transgene does not affect the life cycle of JEV.

Viral genomes exhibit GC content biases shaped by long-term evolution, and foreign sequences with mismatched GC composition may be subject to purifying selection during replication. To assess whether GC modification of the Nluc reporter affects its retention and the replicative fitness of the recombinant viruses over successive replication cycles, each virus was serially passaged 10 times. We then calculated the ratio of luciferase activity to infectious titer (luciferase activity/infectious titer) to assess the stability of luciferase function in each virus. Between JEV-Nluc-WT, JEV-Nluc-L, and JEV-Nluc-H, no significant differences were observed before or after serial passage (P0 and P10) (Fig. 3D). Furthermore, analysis of the Nluc region of the P10 virus by gel electrophoresis revealed no detectable large deletions in any of the recombinant viruses following serial passage (Fig. S1). These findings suggest that the Nluc was stably maintained in each virus during serial passaging.

### Generation and characterization of transgenes with varying GC content in the virus with low GC content

**Fig 4:**
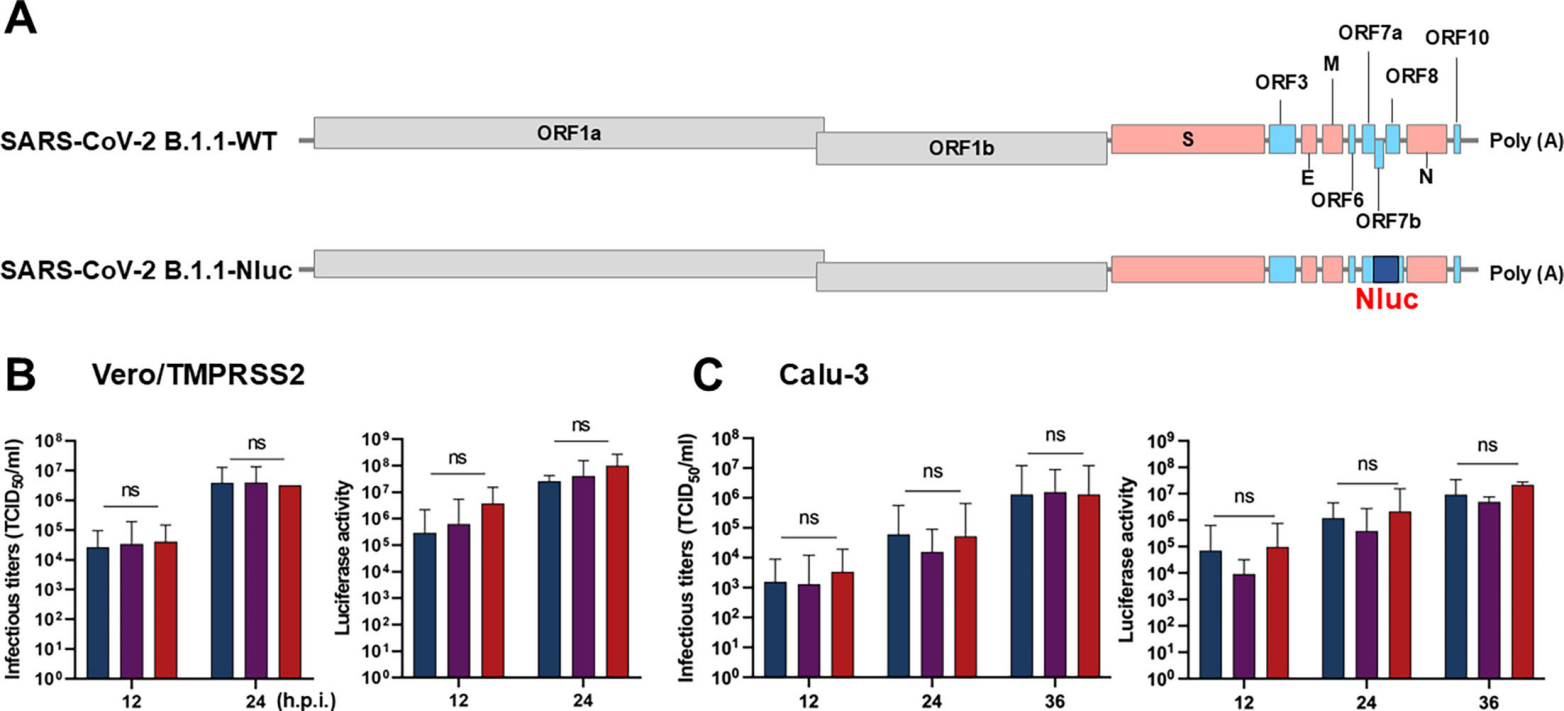
Generation and characterization of the GC-modified Nluc in SARS-CoV-2. (**A**) A schematic representation of the wild-type SARS-CoV-2 (B.1.1) and the recombinant virus carrying Nluc luciferase gene in place of ORFs 6–8. (**B**) *In vitro* growth kinetics of the SARS-CoV-2-Nluc-WT, -L, and -H. VeroE6/TMPRSS2 cells were infected with each of the SARS-CoV-2-Nluc variants (MOI = 0.01). Infectious titers were assessed in the culture supernatants (left panel), and Nluc luciferase activity in the cells (right panel) (*n* = 2). (**C**) *In vitro* growth kinetics of the SARS-CoV-2-Nluc-WT, -L, and -H. Calu-3 cells were infected with each of the SARS-CoV-2-Nluc variants (MOI = 0.1). Infectious titers were assessed in the culture supernatants (left panel), and Nluc luciferase activity in the cells (right panel) (*n* = 2).

We next turned to SARS-CoV-2, which has the lowest GC content and the least stable predicted RNA secondary structure among the three viruses examined here (Fig. 1C). Given this structural context, we hypothesized that GC-rich Nluc variants—which exhibit the highest structural complexity in our analysis (Fig. 1D and Table 1)—might be the most disruptive when inserted into the SARS-CoV-2 genome. We generated recombinant SARS-CoV-2 carrying either Nluc-WT, Nluc-L, or Nluc-H within the accessory protein ORFs 6-–8 as previously reported (9) (Fig. 4A). Two cell lines, VeroE6/TMPRSS2 cells (18) and human lung adenocarcinoma-derived Calu-3 cells, were respectively infected with each of the resulting viruses (Fig. 4B and C). In VeroE6/TMPRSS2 cells, the infectious titers of the culture supernatants (Fig. 4B, left panel) and intracellular luciferase activities (Fig. 4B, right panel) were comparable over time, without significant differences depending on the Nluc GC content. Similarly, no significant differences were observed in the infectious titers and luciferase activity in Calu-3 cells (Fig. 4C). These results indicated that the GC content of Nluc did not affect viral replication or production of infectious particles *in vitro*.

**Fig 5:**
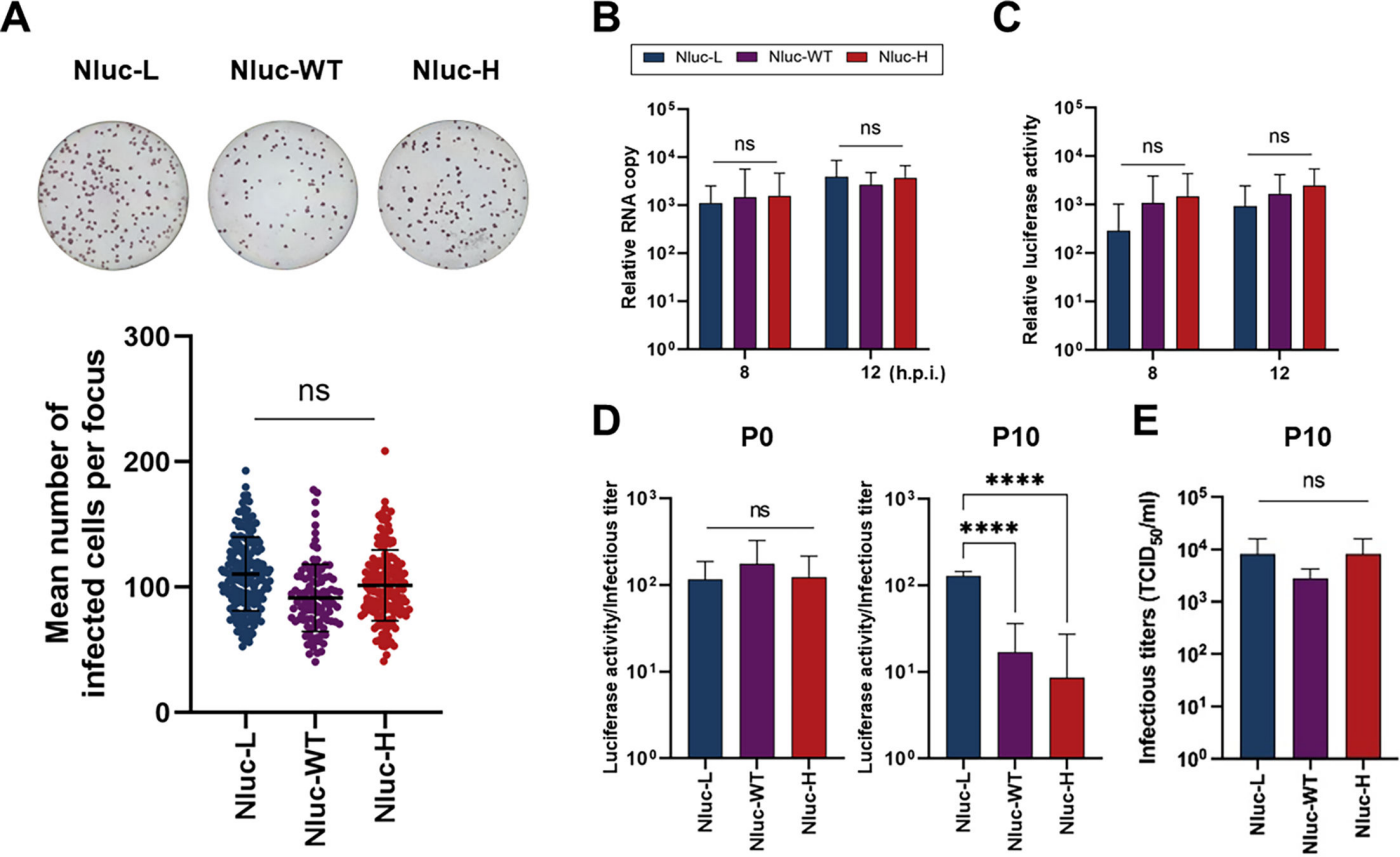
Effects of GC-modified Nluc on replication and genetic stability in SARS-CoV-2. (**A**) The infected cell numbers per FFU were calculated at 30 hpi of SARS-CoV-2-Nluc-WT, -H, and -L. The area of each FFU (upper images are representative for the analysis) was measured for all analyzed FFUs (>100 FFUs per condition) and shown as the scatter plot and mean. (**B**) Viral RNA copy numbers of SARS-CoV-2-Nluc-WT, -L, and -H at 8 and 12 h were normalized to the corresponding values at 4 h (*n* = 3). (**C**) Luciferase activity of SARS-CoV-2-Nluc-WT, -L, and -H at 8 and 12 h was normalized to the corresponding values at 4 h (*n* = 3). (**D**) Ratio of luciferase activity to infectious titer for each of the recombinant SARS-CoV-2-Nluc variants at P0 vs P10 at 9 hpi (*n* = 2). (**E**) Infectious titers of the recombinant SARS-CoV-2-Nluc at P10, measured at 9 hpi (*n* = 2).

Then, we performed the same experimental analyses as those conducted for JEV: no significant effects of the GC modification in the Nluc gene were observed in viral entry/spreading (Fig. 5A), RNA replication (Fig. 5B), or translation (Fig. 5C).

To evaluate the stability of inserted Nluc in each virus, we serially passaged viruses in VeroE6/TMPRSS2 cells for ten rounds to examine the genetic stability of the respective reporters. The ratio of luciferase activities to infectious titers did not significantly differ across viruses at P0 (Fig. 5D, left panel). In contrast, at passage 10, the ratio for SARS-CoV-2-Nluc-L was significantly higher than that of SARS-CoV-2-Nluc-WT or SARS-CoV-2-Nluc-H (Fig. 5D, right panel). Nevertheless, infectious titers at P10 did not significantly differ among the viruses (Fig. 5E). These data suggest that the Nluc-WT and Nluc-H were destabilized during serial passaging, resulting in reduced luciferase activity.

### Characterization of mutations arising during serial passaging of SARS-CoV-2-Nluc

**Fig 6:**
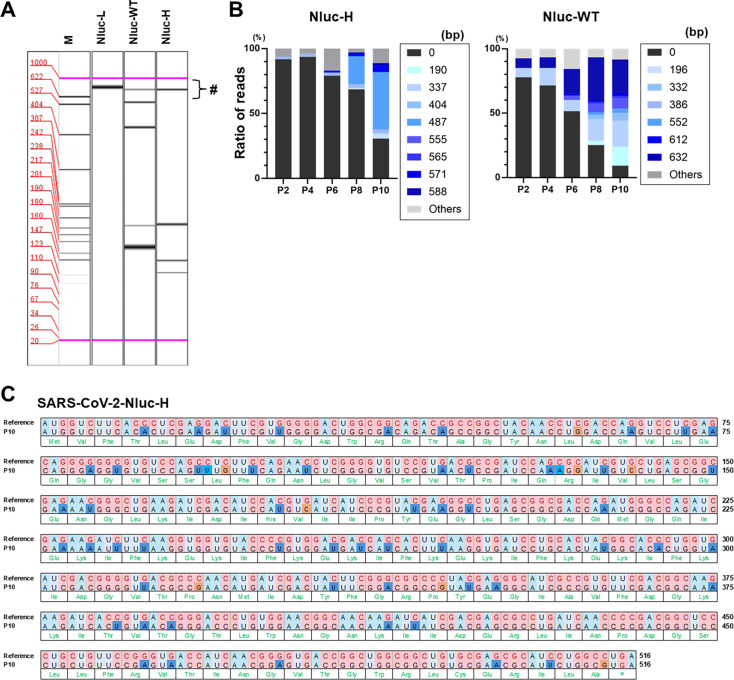
Mutational analysis of GC-modified Nluc in SARS-CoV-2 during passaging. (**A**) Capillary gel electrophoresis of PCR amplicons of the Nluc region purified from the recombinant SARS-CoV-2 viruses after 10 rounds of passaging. M indicates DNA marker as reference; # indicates the full-length Nluc PCR amplicon. The expected PCR product sizes were 757 bp for the full-length Nluc-containing fragment. (**B**) Analysis of the long-read nanopore sequencing of the Nluc-H (left panel) and Nluc-WT (right panel) recombinant SARS-CoV-2 at passages (P) 2, 4, 6, 8, and 10 at the Nluc and its flanking region. Dark gray indicates the proportion of reads that are almost identical to the original sequence, and light gray represents the total proportion of reads classified as “Others” that each contributed <2%. Colors other than gray indicate sequences with deletions, and identical colors correspond to reads of the same sequence. The figure legend indicates the bp length of the deletion represented by each color. (**C**) The upper line shows the original Nluc-H coding sequence (“Reference”) and the lower line displays the same region after 10 serial passages of SARS-CoV-2 -Nluc-H in cell culture (P10).

Because our serial-passage experiments suggested differential reporter stability among the SARS-CoV-2 constructs, we further analyzed the integrity of the inserted Nluc genes by capillary gel electrophoresis (Fig. 6A). Then, to determine the deletion changes during serial passaging, long-read nanopore sequencing in the Nluc region and its flanking regions was conducted. As shown in Fig. 6B, various deletions emerged in SARS-CoV-2-Nluc-WT and SARS-CoV-2-Nluc-H during serial passaging. In addition, the proportion of the full-length Nluc gene (dark gray bars in Fig. 6B) tended to decrease with passaging, falling from 91.8% (P2) to 30.7% (P10) for Nluc-H and from 77.9% to 9.3% for Nluc-WT.

To further investigate the stability of Nluc in each virus, we restricted our analysis to the long-read nanopore sequencing reads of the GC-modified Nluc recombinant SARS-CoV-2 that did not contain deletions in passage 10, we defined a haplotype as a unique full-length sequence of a viral genome segment within the quasispecies and only haplotypes present at a frequency of ≥0.5% were included in the analysis. In SARS-CoV-2-Nluc-H, we detected a haplotype at a frequency of 3.9% (Fig. S2A); positions of the substitutions are shown in Fig. 6C. Within the Nluc region of SARS-CoV-2-Nluc-H, we found a total of 64 substitutions: 54 were G/C to A/U substitutions at the third codon position, 2 were C to A/U substitutions at the first codon position, and 8 were G/C substitutions at the third codon position. Collectively, these mutations decreased the GC content of the viral sequence. In contrast, no mutant haplotypes above the threshold were detected in Nluc-WT or Nluc-L. These results suggest that GC-mismatched Nluc acquired substitutions that shifted its sequence toward the GC content characteristic of SARS-CoV-2 during serial passaging.

### Association between N protein binding efficiency and reporter gene deletions

**Fig 7:**
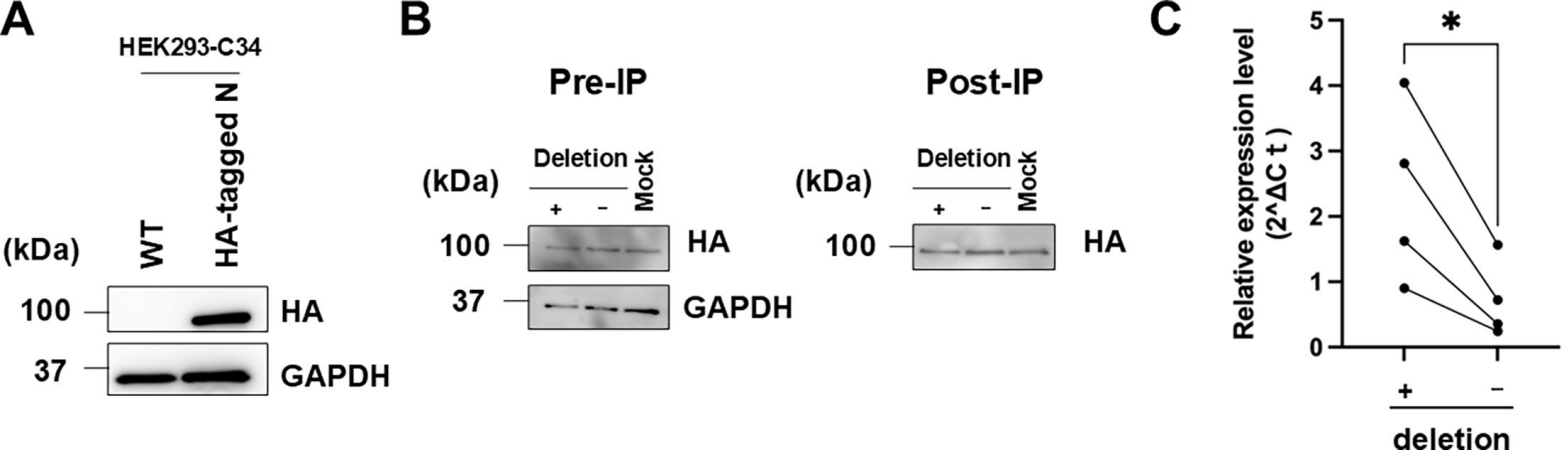
Effects of the Nluc gene on RNA binding to SARS-CoV-2 N. (**A**) Immunoblotting conducted to confirm expression of SARS-CoV-2 N protein in the cells used for the RIP assay. (**B**) Immunoblotting of input (pre-IP) and immunoprecipitated (post-IP) fractions was conducted to ensure quantitative comparability. (**C**) RNA immunoprecipitation assay to compare the RNA binding efficiency of SARS-CoV-2 N protein for the original viral RNA vs that with the deletion mutant observed at passage 10 (*n* = 4).

As previously reported, the SARS-CoV-2 nucleocapsid (N) protein binds viral RNA to facilitate replication and viral particle assembly (19). To elucidate how Nluc deletions affect the interaction between viral RNA and the N protein, we compared the RNA-binding efficiency of each mutant virus to the N protein by immunoprecipitation. The binding efficiency of the N protein to the virus was examined as previously described (20). We established cells expressing the viral N protein fused with the HA-tag by lentiviral transduction (Fig. 7A). In the immunoprecipitation assay, the HA-tagged N protein was detected in both the input and immunoprecipitated fractions, and GAPDH was detected in the input fraction to confirm sample loading (Fig. 7B). The RNA carrying the full-length of Nluc-H showed significantly reduced co-precipitation with the N protein compared to the truncated form that emerged at P10 (Fig. 7C). The restoration of N-RNA co-precipitation in the truncated form provides a functional explanation for the selective loss of the reporter during serial passaging.

### Characterization of transgenes with varying GC content in SARS-CoV-2 *in vivo*

**Fig 8:**
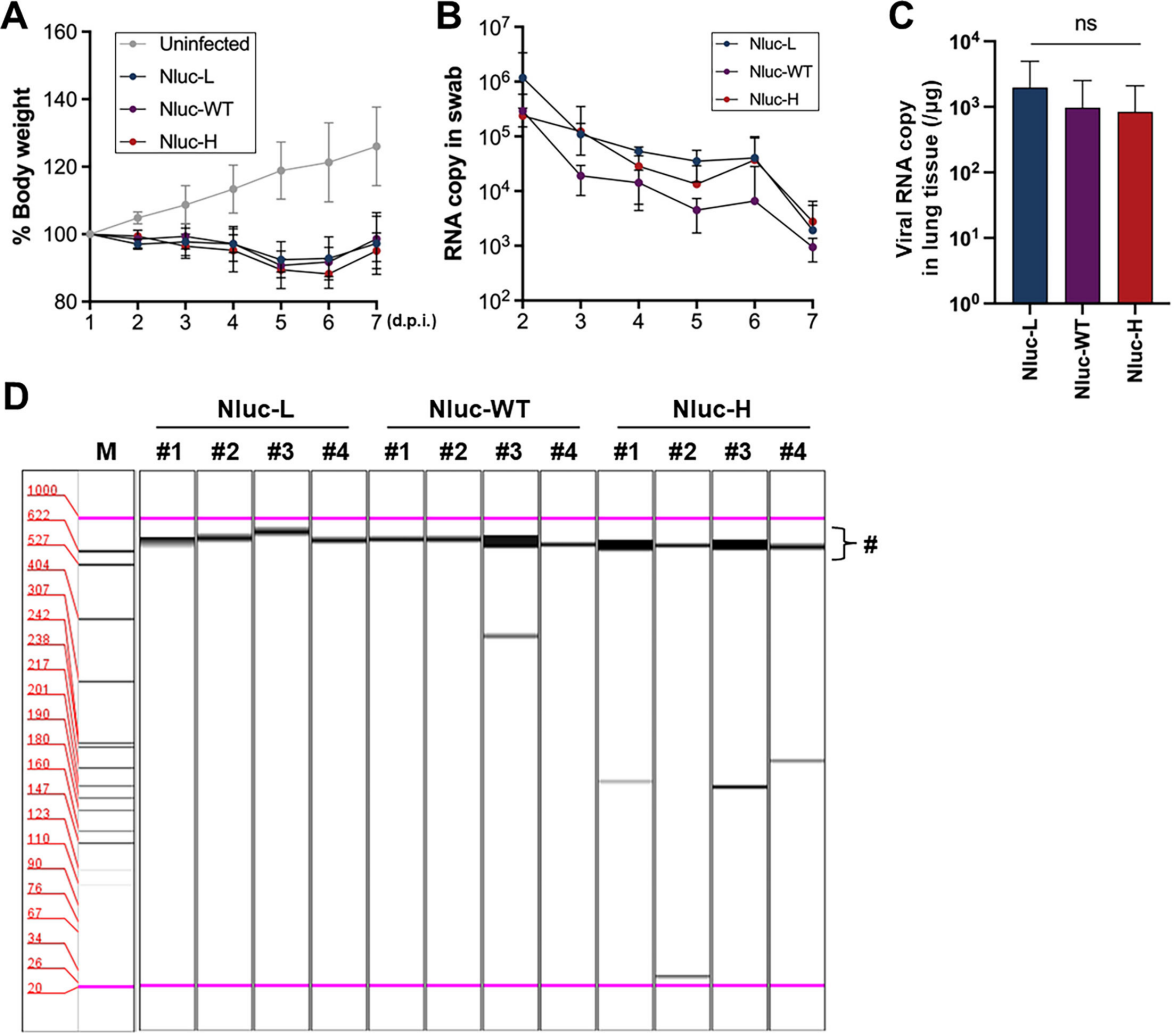
*In vivo* experiment of GC-modified Nluc recombinant SARS-CoV-2. (**A**) Syrian hamsters (*n* = 4 per group) were intranasally inoculated with SARS-CoV-2-Nluc, SARS-CoV-2-Nluc-H, or SARS-CoV-2-Nluc-L, and body weight was monitored every day until 7 dpi. (**B**) Viral RNA copies in the oral swab were measured every day from 1 to 7 dpi. (**C**) The lungs of each hamster were harvested at 7 dpi and viral RNA quantified. (**D**) Hamsters (*n* = 4 per virus) were infected with each of the GC-modified Nluc recombinant SARS-CoV-2 viruses. Viral RNA was isolated from lung tissue at 7 dpi, and capillary gel electrophoresis of PCR amplicons of the Nluc region was performed. Each lane represents a different animal. M indicates DNA marker as reference; # indicates the full-length Nluc PCR amplicon. The expected PCR product sizes were 757 bp for the full-length Nluc-containing fragment. Deletion proportions estimated from the relative peak areas are indicated above each lane in animals where deletion bands were detected (see Results for individual values).

To evaluate the effects of GC mismatch between the transgene and viruses *in vivo*, we conducted experimental infections in hamsters (*n* = 4 per group) using the different recombinant SARS-CoV-2 carrying Nluc. Differences in the GC content of Nluc resulted in no significant differences in viral pathogenicity *in vivo*, as evaluated by changes in body weight (Fig. 8A) and by viral RNA loads in oral swabs from 2 to 7 dpi (Fig. 8B) and in the lungs at 7 dpi (Fig. 8C).

Next, to assess whether the GC content of Nluc influenced reporter stability *in vivo*, capillary gel electrophoresis was performed on the Nluc region amplified from lung tissue at 7 dpi (Fig. 8D). Deletion variants were detected in all four Nluc-H–infected animals, with estimated proportions of 9.7%, 35.5%, 33.3%, and 20.9% in animals #1–#4, and in one of four Nluc-WT–infected animals (18.5% in animal #3). In contrast, no deletions were detected in any of the four Nluc-L–infected animals. Consistent with the *in vitro* findings (Fig. 6A and B), deletion of the Nluc region *in vivo* was thus restricted to viruses carrying GC-mismatched (high-GC) reporters and was not observed in any animal infected with the GC-matched Nluc-L. These results indicate that GC mismatch between the transgene and the SARS-CoV-2 genome drives reporter deletion *in vivo* within a single 7-day host passage, although the proportions of deletion-containing variants varied substantially among animals, suggesting that the adaptation through deletion was still in progress at the time of sampling.

### Generation and characterization of transgenes with varying GC content in the virus with high GC content

**Fig 9:**
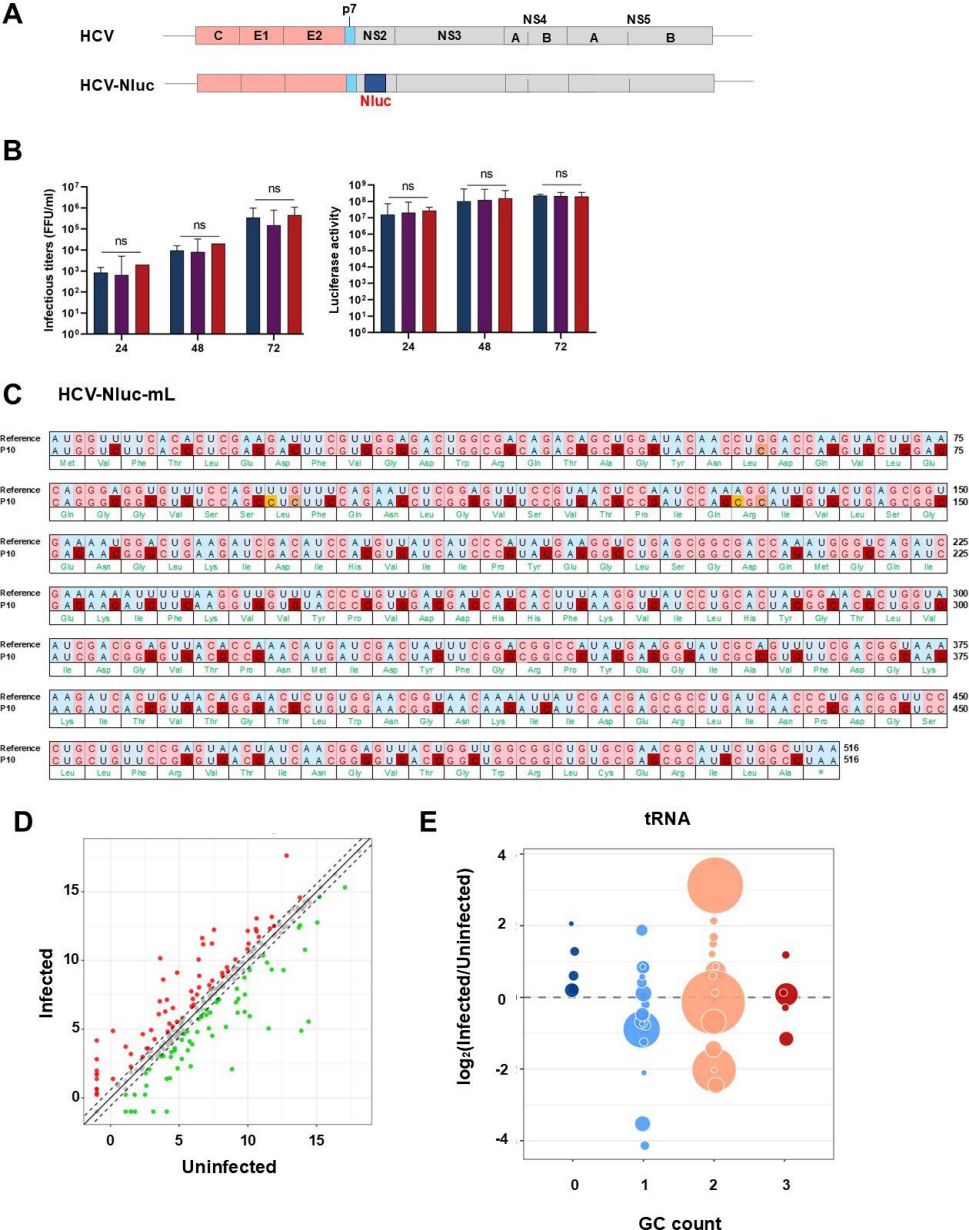
Generation and characterization of GC-modified Nluc in HCV, and alterations in tRNA expression upon HCV infection. (**A**) The gene structures of wild-type HCV and HCV carrying Nluc luciferase gene in place of NS2 protein. (**B**) *In vitro* growth kinetics of HCV-Nluc-WT, -mL, and -H of P10. Huh7.5.1 cells were infected with each of the P10 HCV-Nluc variants (MOI = 0.01). Infectious titers were assessed in the culture supernatants (left panel), and Nluc luciferase activity in the cells (right panel) (*n* = 2). (**C**) The upper line shows the original Nluc-mL coding sequence (“Reference”) and the lower line displays the same region after 10 serial passages of HCV-Nluc-mL in cell culture (P10). (**D**) tRNA isodecoder expression in naive and HCV-infected cells was analyzed by tRNA-seq. Each tRNA isodecoder is plotted as a dot. Red dots indicate isodecoders that are upregulated in HCV-infected cells, and green dots indicate isodecoders that are downregulated (*n* = 2). (**E**) tRNA-seq analysis illustrating the relationship between tRNA GC content (*x*-axis) and expression changes in HCV-infected relative to uninfected cells, calculated as log_2_(infected/uninfected) (*y*-axis). Each circle represents an individual tRNA; circle size is proportional to read frequency, and color reflects GC content (blue = low GC content, red = high GC content). The dotted line indicates an infected/uninfected ratio of 1 (log_2_ = 0).

Finally, we examined HCV, which has the highest GC content and the most stable predicted RNA structure among the three viruses (Fig. 1C). The JFH-1 strain was used throughout, and each Nluc was inserted into the N-terminal region of NS2 (Fig. 9A). Based on the structural framework established in Fig. 1, we predicted that HCV would impose the strongest selective pressure against GC-mismatched (low-GC) Nluc variants. However, despite three independent attempts, we were consistently unable to generate HCV-Nluc-L. Therefore, we synthesized another mutant Nluc gene, designated Nluc-mL, with a GC content of 46% and thus between those of Nluc-L and Nluc-WT. We observed no significant difference in the translation efficiency of Nluc-mL compared to the other Nluc constructs (Fig. S3A). The predicted RNA secondary structure of Nluc-mL showed an MFE of −133.6 kcal mol⁻¹ and structural complexity metrics intermediate between Nluc-L and Nluc-WT (Fig. S3B and Table 1), consistent with our overall observation that higher GC content is associated with greater structural complexity.

Consistent with our previous study (21), the infectious titer of HCV-Nluc at P0 was very low; thus, we decided to subject only the P10 virus for further investigation in the subsequent experiments. Similar to the analyses performed for the other RNA viruses, we then analyzed the viral properties of HCV-Nluc-WT, HCV-Nluc-mL, and HCV-Nluc-H, infecting Huh7.5.1 cells. Among the three recombinant viruses, no significant differences in the infectious titers (Fig. 9B, left panel) and luciferase activities (Fig. 9B, right panel) were observed. As for the genome stability, gel electrophoretic analysis of the Nluc region showed no apparent large deletions in the P10 viruses (Fig. S4). These findings suggest that the Nluc was stably maintained in each virus during serial passaging.

We next performed Nanopore long-read sequencing of the Nluc region of the P10 virus to assess the presence of substitution mutations similar to those observed in SARS-CoV-2 (Fig. S2A). Interestingly, in HCV-Nluc-mL, a GC-enriched haplotype with 97 substitutions was detected at a frequency of 10.6% (Fig. S2B). Of these, 91 were A/U to G/C substitutions at the third codon position, 2 were A/U to G/C substitutions at the first codon position, 1 was an A to G substitution at the second codon position, and 3 were G to C substitutions at the third codon position (Fig. 9C). Additionally, a single-nucleotide variant haplotype was detected at the same locus within Nluc in HCV-Nluc-WT, with an A-to-G substitution at nucleotide 227 of the Nluc. The proportion of each haplotype is shown in Fig. S2, and no additional haplotypes ≥0.5% were observed in any versions of Nluc. These results suggest that, during serial passaging, substitution mutations accumulated predominantly at the third codon position in GC-mismatched Nluc, shifting its GC content toward that of HCV.

Given the pronounced third-position bias, which suggested affecting codon usage, we then assessed the association between wobble at the third codon position and the tRNA environment, conducting tRNA-seq in both naïve and HCV-infected cells. The scatter plots and hierarchical clustering of differentially expressed tRNA isodecoders are shown in Fig. 9D. The expression level of the tRNA isodecoders was altered between the infected vs naïve cells. Applying a 1.5-fold change threshold revealed extensive alterations in tRNA isodecoder expression, affecting 147 of 255 species (57.6%), with comparable numbers of upregulated (*n* = 77) and downregulated (*n* = 70) isodecoders. Calculating the GC content of total tRNA isodecoders, the GC content of HCV-infected cells was 59.9% vs 57.9% in uninfected cells (Fig. 9E). Taken together, these findings suggest that HCV infection remodels the cellular tRNA pool toward a more GC-rich composition.

## DISCUSSION

Although GC content varies widely among viruses, its functional significance has remained elusive. By employing recombinant viruses engineered with foreign genes of distinct GC contents, we demonstrate here, for the first time to our knowledge, the critical role of GC content in shaping viral genome stability and propagation in the RNA viruses examined in this study.

The structural analyses presented in Fig. 1C and D and Table 1 establish a framework for interpreting the contrasting passage outcomes observed in the three viruses. Higher GC content was consistently associated with greater predicted RNA structural stability and complexity, both at the level of viral genomes and at the level of Nluc variants. Within this framework, the deletion-prone behavior of GC-mismatched Nluc in SARS-CoV-2 (Fig. 6) is consistent with the relatively low structural stability of the SARS-CoV-2 genome, in which insertion of a structurally complex GC-rich foreign sequence may impose disproportionate constraints. Conversely, the more structurally stable genomes of HCV and JEV may better accommodate insertions through sequence-level adjustment rather than deletion.

First, we used JEV as a virus with moderate GC content. In JEV, the GC content of Nluc did not affect the infectious titers and luciferase activity both in Huh7 cells and C6/36 cells (Fig. 2B and C). To evaluate the effects of the GC modification in each step of JEV propagation, such as viral entry, gene replication, and translation, we analyzed the number of infected cells per FFU upon the respective virus infection (Fig. 3A) and the viral copies and luciferase activities in the early infectious phase (Fig. 3B and C). These results showed that there were no significant differences in viral entry/spreading, RNA replication, and translation among the Nluc variants. Furthermore, serial passaging over ten rounds did not result in detectable loss of Nluc function (Fig. 3D), indicating that GC mismatch between the reporter and the JEV genome does not impose sufficient selective pressure to drive reporter loss within this experimental window. Thus, JEV tolerates a broad range of reporter GC content without measurable fitness cost.

Then, we used SARS-CoV-2 as a virus with low GC content and generated SARS-CoV-2 with various GC contents. In SARS-CoV-2, the GC content of Nluc did not affect the infectious titers and luciferase activity both in Vero/TMPRSS2 cells and Calu-3 cells that are commonly used in SARS-CoV-2 research (Fig. 4B and C).

To evaluate each virus in various aspects, we calculated the number of infected cells per FFU (Fig. 5A), viral copies, and luciferase activities in the early infectious phase (Fig. 5B and C). These results showed that there were no significant differences in viral entry/spreading, RNA replication, translational efficiency, and viral spreading in each SARS-CoV-2-Nluc. To examine how GC mismatch of the transgene influences replicative fitness of the recombinant viruses over successive replication cycles, we passaged each virus ten times and found that Nluc-WT and Nluc-H in SARS-CoV-2 had a decrease in luciferase activity to infectious titer ratio (Fig. 5D). These results suggest that genetic stability varies with virus species and the GC content of Nluc.

In SARS-CoV-2, our observations after serial passaging indicated that foreign genes with a high GC content, such as Nluc, and structurally complex features were less stable and were gradually lost, as demonstrated by the accumulation of deletion mutations over time (Fig. 6A and B). SARS-CoV-2 differs from JEV and HCV in several respects that may contribute to the pronounced instability of GC-mismatched transgenes. It possesses a substantially larger genome (~29,895 nt vs ~10,926 nt for JEV and ~9,678 nt for HCV), exhibits lower overall GC content and less stable RNA secondary structures (Fig. 1C), and appears to accommodate deletion-based genome rearrangements more readily than substitution accumulation. Although these characteristics are associated with transgene instability, their individual contributions cannot be fully distinguished in the present study.

Because SARS-CoV-2 possesses a relatively AU-rich and structurally less constrained genome, insertion of a GC-rich foreign gene may have a greater impact on local RNA architecture than in viruses with intrinsically higher GC content and more stable RNA structures. The SARS-CoV-2 N protein is known to associate extensively with viral genomic RNA and plays critical roles in genome packaging and replication. To investigate whether the observed deletions influenced this interaction, we performed an RNA immunoprecipitation (RIP) assay (Fig. 7C), an approach previously used to evaluate SARS-CoV-2 RNA–N protein association (20). Deletion of Nluc enhanced N protein binding, suggesting that the presence of Nluc interfered with efficient RNA–N interactions. Although the RIP assay does not directly measure RNA secondary structure, one possible explanation is that the GC-rich Nluc sequence introduced structurally complex RNA elements that reduced accessibility of viral RNA to the N protein. In the context of the relatively AU-rich and structurally less constrained SARS-CoV-2 genome, such structural perturbations may be particularly detrimental, thereby creating selective pressure for deletion of the foreign sequence. Future studies employing RNA structural analyses, such as SHAPE- or DMS-based probing, together with targeted mutagenesis designed to uncouple GC content from RNA structure, will be required to determine the relative contributions of sequence composition and RNA architecture to N protein binding affinity and transgene stability.

*In vivo* experiments also confirmed that SARS-CoV-2-Nluc exhibited no apparent differences in pathogenicity, as assessed by body weight (Fig. 8A) and viral RNA copy numbers in oral swabs and lung tissue (Fig. 8B and C). Capillary gel electrophoresis of the Nluc region amplified from lung tissue at 7 dpi (Fig. 8D) detected deletion variants in all four Nluc-H–infected animals (estimated proportions of 9.7%, 35.5%, 33.3%, and 20.9% for animals #1–#4) and in one of four Nluc-WT–infected animals (18.5% in #3), whereas no deletions were detected in any of the four Nluc-L–infected animals. The restriction of deletion to GC-rich Nluc variants *in vivo* mirrors the *in vitro* observations (Fig. 6A and B) and is difficult to reconcile with a model in which transgene GC content has no impact on viral fitness in the animal host, because such a model would predict no systematic difference between Nluc-L and Nluc-H/WT in deletion frequency.

However, we acknowledge two important caveats in interpreting these *in vivo* data. First, the deletion proportions in Nluc-H animals (~10–35%) and the detection of deletion in only one of four Nluc-WT animals indicate that adaptation through deletion is partial within a single 7-day host passage, consistent with the gradual accumulation observed from P2 to P10 *in vitro* (Fig. 6B). Second, because the lung tissues from this cohort were entirely consumed for RNA extraction, which was required for the gel analyses presented in Fig. 8D—we could not directly measure Nluc reporter activity in matched lung homogenates. The relative contributions of rapid deletion-based adaptation vs an intrinsic insensitivity of gross pathogenicity readouts (body weight and total viral RNA) to reporter GC content therefore cannot be fully resolved from the current data set. Longitudinal sampling with individual-matched reporter-activity measurements would be informative for future studies.

We then set out to construct recombinant HCV carrying our GC-modified Nluc variants. Although we were readily able to generate SARS-CoV-2 and JEV each containing either Nluc-WT, Nluc-L, or Nluc-H, we were unable to generate HCV-Nluc-L despite repeated attempts. This outcome might reflect the large GC content gap between Nluc-L (GC content = 34%) and the HCV genome (GC content = 58%) and is consistent with our overall observation during this study that GC content plays a critical role in RNA viruses. Consequently, we generated a more moderate form of Nluc-L, termed Nluc-mL (GC content = 46%), and were able to successfully generate this recombinant HCV.

Analysis of HCV-Nluc was limited to the P10 virus because of the low infectious titer at P0; however, no significant differences were observed among the HCV-Nluc within the evaluable range (Fig. 9B).

A more detailed analysis of the long-read nanopore sequencing data for the Nluc region, focusing on substitution mutations in reads without deletions, revealed that SARS-CoV-2-Nluc-H acquired 64 substitutions that reduced the GC content of Nluc (Fig. 6C). Strikingly, 96.9% of them were located at the third nucleotide of each codon and 87.5% of them were G/C to A/U substitutions and the remaining 12.5% were G/C substitutions. Given the potential functional relevance of these changes, we next applied long-read nanopore sequencing to our recombinant JEV- and HCV-Nluc viruses (Fig. S2B and C). In HCV-Nluc-mL, after 10 rounds of serial passaging, we identified a GC-enriched haplotype, with 96.9% of the 97 substitutions located at the third nucleotide of each codon—the position where tRNAs can “wobble” (22). Regarding GC content, 96.9% of the substitutions changed the A or U to G or C, and 3.1% changed G to C (Fig. 9C). To account for the greater number of substitutions in HCV compared to SARS-CoV-2, we hypothesize that differences in replicase fidelity are involved: SARS-CoV-2 benefits from nsp14-mediated proofreading (23), whereas HCV NS5B lacks such activity (24). Notably, SARS-CoV-2 exhibited a high frequency of deletion mutations whereas no deletions were detected in HCV. Taken together, these contrasting features—differences in deletion frequency and replicase proofreading activity—underscore the need for future studies to elucidate the mechanistic basis of these differences.

We hypothesized that the pool of tRNAs within the infected cells might be different from that in uninfected cells, and thus we conducted tRNA-seq (25, 26). tRNA-seq of HCV-infected cells vs uninfected cells showed that the tRNA environment significantly changed upon infection with HCV (Fig. 9D). In a previous report, mimivirus, one of the largest known viruses that was initially discovered infecting amoebae, is prone to exhibit the preference of the environment of GC content in cells (27). The substitutions that increased the GC content of HCV-Nluc-mL are consistent with the gap in GC content between the introduced low-GC reporter and the HCV genome. Our tRNA-seq analysis indicates that the cellular tRNA pool shifts toward a more GC-rich composition following HCV infection. The overall direction of this shift is consistent with the GC-directed substitution bias observed in HCV-Nluc-mL, although we did not find a one-to-one correspondence between individual tRNA isodecoders and the specific codons affected. We therefore consider these tRNA changes to be compatible with, rather than a direct driver of, the observed mutational bias, and propose that the high GC content of HCV is accompanied by a tRNA environment that may favor the emergence of GC-enriched variants.

For JEV, a virus with intermediate GC content, we observed that all versions of Nluc remained stable even after ten serial passages. In contrast, GC-content-biased viruses like HCV and SARS-CoV-2 exhibited frequent mutations when the GC content of the inserted Nluc did not match their own. These results suggest that viruses with intermediate GC content may better tolerate and stabilize foreign genes. However, this hypothesis requires further validation through experiments across diverse viral backgrounds and host systems to determine whether GC compatibility universally influences the genetic stability of inserted sequences.

In summary, our findings advance the understanding of the significance of GC content in RNA viruses analyzed in this study and suggest that similar features should be investigated in other RNA viruses. Previous work showed that codon optimization is essential for stable transgene insertion in viruses (28), and we have also successfully generated recombinant viruses with a GC-optimized reporter gene (9). Here, to the best of our knowledge, we present a mechanistic analysis demonstrating how viral GC content regulates the fate of exogenous sequences. We also offer rational strategies for engineering reporter genes that are more stable in viruses. Further exploration of such assays in other viruses is necessary to improve our understanding of the molecular mechanisms underlying viral replication and pathogenesis.

## MATERIALS AND METHODS

### Cell lines

All mammalian cell lines were cultured at 37°C in a humidified atmosphere with 5% CO_2_. Human hepatocellular carcinoma–derived Huh7.5.1 cells, human embryonic kidney–derived HEK293T, Lenti-X 293T cells (Takara Bio, Shiga, Japan), and human embryonic kidney-C34 cells (29) were maintained in DMEM (high glucose) (Nacalai Tesque, Kyoto, Japan) supplemented with 1% penicillin–streptomycin (PS) (Nacalai Tesque) and 10% fetal bovine serum (FBS). Human hepatocellular carcinoma–derived Huh7 cells were maintained in DMEM (low glucose) (Fujifilm WAKO Chemical, Osaka, Japan) supplemented with 1% PS and 10% FBS. VeroE6/TMPRSS2 cells (VeroE6 cells stably expressing human TMPRSS2; JCRB Cell Bank, JCRB1819) (18) were propagated in DMEM (low glucose) (Fujifilm WAKO Chemical) containing 10% FBS, G418 (1 mg/mL; Fujifilm WAKO Chemical), and 1% PS. Calu-3 cells (ATCC, HTB-55) were maintained in Eagle’s minimum essential medium (EMEM) (Nacalai Tesque) containing 10% FBS and 1% PS. The *Aedes albopictus* mosquito-derived cell line C6/36 was grown in Leibovitz’s L-15 medium (Thermo Fisher Scientific, Waltham, MA, USA) with 10% tryptose phosphate broth, FBS, and 1% PS at 32°C.

### Evaluation of translation efficiency of Nluc reporter vectors in mammalian cells

The wild-type Nluc luciferase gene (GenBank accession number: MZ727726) possessing 53% GC content was synonymously substituted to create three mutants with GC content of 34% (Nluc-L), 46% (Nluc-mL), or 64% (Nluc-H), respectively (Fig. 1A). The three Nluc mutants were synthesized commercially (Fasmac, Kanagawa, Japan). To evaluate translation efficiency in cell culture, the Renilla luciferase gene in the pRL[Rluc/TK] vector (Promega, Madison, WI, USA) was replaced with each of the Nluc mutant variants using the In-Fusion technique (Takara Bio). pGL4.54 [*luc2*/TK] (Promega) was used as a control. HEK293T cells were transfected with 50 ng of the respective vectors, and luciferase activity was measured using a Nano-Glo Dual Luciferase Reporter assay system (Promega) with an AB-2270 Octa luminometer (ATTO, Tokyo, Japan) according to the protocol provided by the manufacturers.

### Generation of recombinant viruses carrying Nluc

To generate SARS-CoV-2 carrying each of the Nluc variants, Nluc-WT in pcDNA.3.1-CoV-2-G9-10-Nluc (GC content: 53%) (9) was replaced with either Nluc-L or Nluc-H using the In-Fusion technique. The recombinant viruses carrying the respective Nluc variants were generated by a circular polymerase extension reaction (CPER) as previously described (30). The CPER product was transfected into VeroE6/TMPRSS2 cells and, after cytopathic effect was observed, the culture supernatants were harvested and stored as the viral stock.

To generate the various JEV-Nluc recombinant viruses, respective mutant Nluc genes were inserted into the sub-genome encoding 198-777 nt of JEV AT31 (GenBank number: AB196923) per a previous study (31). Using the In-Fusion technique, each Nluc was inserted into pCRII-JEV AT31 F1 (21), and the resulting plasmids were designated as pCRII-JEV AT31-Nluc-L, pCRII-JEV AT31-Nluc-WT, or pCRII-JEV AT31-Nluc-H. The recombinant viruses were then generated by CPER as described previously (21). For generation of HCV carrying the different Nluc variants, we employed pHH-JFH1-Nluc (32) as a template and replaced the wild-type Nluc gene with either Nluc-mL or Nluc-H using the In-Fusion technique, and recovered infectious recombinant viruses as previously described (32, 33). Huh7.5.1 cells were used with TransIT-LT1 according to the manufacturer’s protocol. The culture supernatants were harvested and used for the downstream experiments. Because the infectious titers obtained from the supernatants of cells transfected with the viral cDNA clones were extremely low (at passage 0 [P0]), we used the virus collected after 10 serial passages (P10) for subsequent experiments.

The infectious titers of the prepared working viruses were measured by a focus-forming unit (FFU) assay for HCV and JEV with the respective antibodies as previously reported (21); and 50% tissue culture infectious dose (TCID_50_) for SARS-CoV-2. Rabbit anti-HCV NS5A antibody was generated as previously described (34) and was used for immunostaining of HCV-infected cells. Anti-4G2 rabbit monoclonal antibody was purchased from Absolute Antibody (Redcar, UK) for JEV. Alexa Fluor (AF) 488-conjugated anti-rabbit IgG antibodies were obtained from Thermo Fisher Scientific. The value of TCID_50_/mL was calculated using the Reed–Muench method (35). Luciferase activity of the recombinant viruses was measured using a Nano-Glo Luciferase Reporter assay system (Promega).

All recombinant viruses were stored at −80°C until use. The viral sequence of all recombinant viruses was determined by the Sanger method with SeqStudio Genetic Analyzer (Thermo Fisher Scientific) and/or an outsourced service (FASMAC). The primers used in this study can be provided upon request.

### Capillary electrophoresis

Multiplex PCR amplifications were profiled on a Qsep 1-Plus (BiOptic, New Taipei City, Taiwan) with the S1 cartridge under the “HR-DNA-20” quick method (injection 4 kV/8 s; run 6 kV/3 min). Lower (20 bp) and upper (1,000 bp) markers were co-injected to ensure run-to-run accuracy. Peaks were automatically called and visualized by Q-Analyzer (“Peak-Calling” function). The proportion of viral populations carrying Nluc deletions in lung tissue samples (Fig. 8D) was estimated from the relative peak areas of the full-length and deletion-containing amplicons within each lane.

### RNA immunoprecipitation (RIP)

The cDNA of the SARS-CoV-2 N gene was inserted between the XhoI and XbaI sites of the lentiviral vector pCSII-EF-RfA using the In-Fusion technique, and the resulting plasmid was designated as pCSII-EF-SARS-CoV-2-N-HA. The lentiviral vectors and ViraPower lentiviral packaging mix (Thermo Fisher Scientific) were co-transfected into HEK293-C34 cells, and the supernatants were recovered at 48 h post-transfection (36).

RIP was performed using the RIP-Assay kit (MBL, Tokyo, Japan) following the manufacturer’s protocol. HEK293-C34 cells were lentivirally transduced with the HA-tagged SARS-CoV-2 N protein. Expression was confirmed by immunoblotting. Lysates from the HA-tagged N-protein expressing HEK 293 cells were incubated with anti-HA-tag mAb-Magnetic Beads (MBL). The immunoprecipitated RNA was analyzed by RT-qPCR using Power SYBR Green RNA-to-Ct 1-step Kit (Thermo Fisher Scientific). Gene-specific primers targeting the ORF7b region of SARS-CoV-2 were used (pair—Fwd 5′-GTGACTCACGTTTTGCTGCA-3′, Rev 5′-TGTCACTGCTACTGGAATGGT-3′).

### Long-read nanopore sequencing

Viral RNA was extracted using the PureLink RNA Mini kit (Thermo Fisher Scientific). The amplicons were individually purified using the AMPure-XP bead (Beckman, Brea, CA, USA) following the manufacturer’s protocol. The amplicon DNA was end-repaired and A-tailed with the NEBNext Ultra II End Repair/dA-Tailing Module (E7546, NEB), then barcoded with the Native Barcoding Kit 96 V14 (SQK-NBD114.96; Oxford Nanopore Technologies, Oxford, UK) according to the manufacturer’s protocol. A total of 45 fmol was loaded onto R10.4.1 MinION flow cells (FLO-MIN114, Oxford Nanopore Technologies). Sequencing was performed on a MinION Mk1C (MinKNOW version 24.02.5, live base-calling off). Raw pod5 files were base-called by super accuracy base-calling with Dorado (version 0.9.0).

Raw FASTQ files were imported into CLC Genomics Workbench ver. 23.0.5 (QIAGEN, Venlo, the Netherlands). Primer sequences were removed and bases with a Phred score <20 were trimmed using the Trim Reads tool (default parameters). Trimmed reads were mapped to the reference genome with Map Long Reads to Reference (default parameters). Variants present at ≥2% allele frequency were identified with Basic Variant Detection, using the parameter Minimum variant quality = 30.

### Calculation of viral haplotype composition and sequences within a quasispecies population

In viral quasispecies, a haplotype is defined as a nucleotide sequence representing an individual viral genome within a heterogeneous population. To characterize both the composition and sequence of haplotypes in the viral population after 10 serial passages (P10 virus), reads obtained by nanopore sequencing as described above were primer-trimmed using Cutadapt and filtered to retain only those within ±5 bp of the consensus length. The trimmed reads were realigned to each consensus sequence using Indelfixer, and haplotypes were reconstructed using QuasiRecomb. Haplotypes with an estimated frequency <0.5% were excluded from reporting.

### tRNA-seq

Total RNA collected from the naïve Huh7.5.1 cells and the cells infected with HCV carrying the low GC mutant of Nluc was purified using the miRNeasy Mini kit (QIAGEN, Hilden, Germany) according to the manufacturer’s protocol. Pre-treatment of RNA, library preparation, next-generation sequencing, and some aspect of data analysis were performed by a commercial service (Arraystar, Rockvile, MD, USA). In brief, full-length tRNA sequencing and tRNA fragment sequencing were performed separately to provide more accurate size selection and better sequencing depth and coverage. For full-length tRNA sequencing, 5 µg of total DNase-treated RNA was resolved on a urea-polyacrylamide gel and recovered within a size window of 60–100 nt and then pre-treated with demethylation enzymes (25) to prepare for efficient reverse transcription and adapter ligation. After the pre-treatment, the resulting fragments of ~19–35 nt were converted to small RNA sequencing libraries using NEBNext Multiplex Small RNA Library Prep Set (Illumina, San Diego, CA, USA) and quantified on an Agilent Bioanalyzer before 50 bp single-end sequencing on an Illumina NextSeq 500 instrument.

Sequencing quality was assessed with FastQC, and raw sequencing read data that passed the Illumina chastity filter was used for further data analysis, including alignment of trimmed reads (cutadapt) to mature tRNA reference sequences from GtRNAdb (37) and mitotRNAdb (38) using BWA. For tRNA alignment, the maximum number of mismatches was two. tRNA expression levels were measured with tag count, and the multi-map-corrected number of reads overlapping the tRNA (mrcount) was then calculated. For differential expression analysis, we used the R package edgeR, with a fold change cutoff of 1.5 and a requirement that CPM ≥ 20 (mean in one group).

### Viral replication kinetics

*In vitro* growth kinetics of recombinant viruses carrying the wild-type or mutant Nluc variants were evaluated in susceptible cell lines. In the case of the recombinant HCVs, Huh7.5.1 cells were inoculated at a multiplicity of infection (MOI) of 0.01. The cells and supernatants were collected at 24, 48, and 72 hpi and used to measure luciferase activity in the cells and to titer infectious viruses in the supernatants. The replication kinetics of the recombinant JEVs were determined in Huh7 inoculated at an MOI of 0.01 and in C6/36 cells inoculated at an MOI of 0.001, respectively. The cells and supernatants were collected at 12, 24, and 36 hpi for Huh7 cells and at 24 and 36 hpi for C6/36 cells. The replication kinetics of recombinant SARS-CoV-2 were evaluated in VeroE6/TMPRSS2 cells inoculated at an MOI of 0.01 and in Calu-3 cells inoculated at an MOI of 0.1. The cells and supernatants were collected at 12 and 24 hpi for VeroE6/TMPRSS2 cells and at 12, 24, and 36 hpi for Calu-3 cells.

### Quantitative RT-PCR

For quantification of viral RNA copies, total RNA was extracted from cells by using a PureLink RNA Mini Kit (Thermo Fisher Scientific), and then first-strand cDNA synthesis and qRT-PCR were performed by using a TaqMan RNA-to-C_T_1-step Kit and ViiA7 system (Thermo Fisher Scientific), respectively, according to the manufacturer’s protocols. For quantification of viral RNA of JEV, the primer sets for the detection of the noncoding region reported in the previous studies (39) were used. The viral RNA load of SARS-CoV-2 in the oral swabs and respiratory tissues was determined by quantitative RT–PCR (qRT-PCR). For quantification of viral RNA copies, total RNA was extracted from specimens using a PureLink RNA Mini Kit (Thermo Fisher Scientific), and then first-strand cDNA synthesis and qRT-PCR were performed using One Step PrimeScript III RT-PCR Kit (Takara Bio) and QuantStudio 5 Real-Time PCR System (Thermo Fisher Scientific), respectively, according to the manufacturer’s protocols. For quantification of viral RNA, primer sets detecting the region encoding part of the viral nucleocapsid phosphoprotein as reported in a previous study were used (40). Fluorescent signals were determined by the QuantStudio 5 Real-Time PCR System.

### Focus-forming assay

Focus-forming assay was performed as previously described (40). Briefly, 1 day before infection, Huh7 or VeroE6/TMPRSS2 cells were seeded into a 24-well plate and infected with each virus at 37°C for 1 h. A mounting solution containing 3% FBS and 1.5% or 1% carboxymethyl cellulose (Sigma-Aldrich) was overlaid, followed by incubation at 37°C. At 48 or 30 hpi, the culture medium was removed, and the cells were washed with PBS three times and fixed with 4% paraformaldehyde phosphate. A mouse anti-SARS-CoV-2 N monoclonal antibody (R&D Systems, Minneapolis, MN, USA) was used as the primary antibody for SARS-CoV-2. For JEV detection, 4G2 was used as the primary antibody. After washing, the cells were incubated with HRP-conjugated anti-mouse IgG and visualized using the VECTASTAIN ABC-HRP Kit, Peroxidase (Mouse IgG), and the Vector VIP Substrate Kit, Peroxidase (HRP) (Vector Laboratories, Newark, CA, USA). The stained cells were washed with tap water and dried, and images of stained plaques were acquired using a BZ-X810 fluorescence microscope and BZ-X800 Analyzer (Keyence, Osaka, Japan). The area of each FFU was measured for all FFUs analyzed (>100 FFUs per sample), and the number of cells per FFU was then estimated by dividing the FFU area by the mean area per cell calculated from five representative FFUs. The area of each focus was measured using Fiji/ImageJ version 2.9.0.

### *In vitro* virus passages

VeroE6/TMPRSS2 cells were inoculated with each of the three versions of SARS-CoV-2-Nluc at an MOI of 0.01. At 2 days post-infection (dpi), culture supernatants were collected, and 200 µL of these were used to inoculate naïve VeroE6/TMPRSS2 cells. This procedure was serially repeated 10 times. The culture supernatants were collected to assess viral titers and for viral sequencing.

Huh7.5.1 cells inoculated with the three recombinant HCVs, each carrying the respective Nluc, were passaged every 3 days. This procedure was serially repeated in total 10 times. The culture supernatants were collected to assess viral titers and for viral sequencing. Huh7 cells were inoculated with the three versions of JEV-AT31-Nluc at an MOI of 0.01. After 2 dpi, culture supernatants were collected, and 200 µL of these were used to inoculate naïve Huh7 cells. This procedure was serially repeated ten times. The culture supernatants were collected to assess viral titers and for viral sequencing.

### Immunoblotting

Cells lysed on ice in lysis buffer (20 mM Tris-HCl [pH 7.4], 135 mM NaCl, 1% Triton X-100, and 10% glycerol) supplemented with a protease inhibitor cocktail, cOmplete Mini (Roche), were boiled in loading buffer and subjected to 5–20% gradient SDS-PAGE. The proteins were transferred to polyvinylidene difluoride membranes (Millipore, Burlington, MA, USA). The membranes were incubated with an anti-HA antibody (BioLegend, San Diego, CA, USA), followed by incubation with horseradish peroxidase (HRP)-conjugated secondary antibodies (Jackson ImmunoResearch Laboratories, West Grove, PA, USA). GAPDH was detected using an HRP-conjugated anti-GAPDH antibody (Fujifilm Wako Chemical). The immune complexes were visualized with SuperSignal West Femto substrate (Thermo Fisher Scientific) and detected by use of a LuminoGraph I analyzer (Atto).

### Animal experiments with SARS-CoV-2

Experimental infection with SARS-CoV-2 in the hamster model was performed as previously described (12, 30, 41–45). Syrian hamsters (male, 4-week-old) were purchased from Japan SLC Inc (Shizuoka, Japan). For the virus infection, animals were anesthetized by intramuscular injections with a mixture of 0.15 mg/kg medetomidine hydrochloride (Domitor: Koriyama, Nippon Zenyaku Kogyo, Fukushima, Japan), 2.0 mg/kg midazolam (Dormicum: Fujifilm WAKO Chemical), and 2.5 mg/kg butorphanol (Vetorphale: Meiji Seika Pharma, Tokyo, Japan) or 0.15 mg/kg medetomidine hydrochloride, 4.0 mg/kg alphaxalone (Alfaxan: Meiji Animal Health, Kumamoto, Japan), and 2.5 mg/kg butorphanol. Then, hamsters were intranasally inoculated with 100 μL of each virus or 100 μL of saline. Oral swabs were collected at the indicated time points. Body weight was recorded daily through 7 dpi. Lung tissues were anatomically collected at 7 dpi. The viral RNA load in the oral swabs and respiratory tissues was determined by quantitative RT–PCR (qRT-PCR). For quantification of viral RNA copies, total RNA was extracted from specimens using a PureLink RNA Mini Kit (Thermo Fisher Scientific), and then first-strand cDNA synthesis and qRT-PCR were performed using One Step PrimeScript III RT-PCR Kit (Takara Bio) and QuantStudio 5 Real-Time PCR System (Thermo Fisher Scientific), respectively, according to the manufacturer’s protocols.

### Calculation of MFE

Using the ViennaRNA package (15), the MFE of each virus was calculated. To account for the length dependency of MFE, we applied a sliding-window analysis (window = 300 bp, step = 1 bp) and calculated the MFE for each window.

### Quantification and statistical analysis

Results are expressed as the means ± 95% confidence intervals. Statistical significance was determined using Student’s *t*-test or analysis of variance (ANOVA), as appropriate, with GraphPad Prism (Boston, MA, USA) (Software ver. 10.4.1). All *in vitro* experiments were conducted in at least two independent replicates. Significantly different values (*P*<0.05) are indicated by an asterisk.

## Data Availability

Raw sequencing reads for Nluc regions in viruses (Fig. 6 and 9 and Fig. S2) have been deposited in the NCBI Sequence Read Archive (SRA) under BioProject accession PRJNA1379292. Raw sequencing data for the tRNA-seq experiments (Fig. 9) have been deposited under BioProject accession PRJNA1379518.

## SUPPLEMENTAL MATERIAL

Supplemental figures jvi.00882-26-s0001.pdf Fig. S1 to S4.

ASM does not own the copyrights to Supplemental Material that may be linked to, or accessed through, an article. The authors have granted ASM a non-exclusive, world-wide license to publish the Supplemental Material files. Please contact the corresponding author directly for reuse.
