# Supplementary material for: GC content mismatch of transgene destabilizes RNA virus genomes
Source: J Virol. 2026 Jul 13;100(8):e00882-26. doi: 10.1128/jvi.00882-26 (PMC13483392; doi:10.1128/jvi.00882-26)

Supplementary Figure lengends

Figure S1. Gel electrophoresis of the recombinant JEVs after ten rounds of serial passage. Gel electrophoresis of PCR amplicons of the Nluc region purified from the recombinant JEVs after ten rounds of passaging. M indicates DNA marker as reference. The expected PCR product sizes were 1,052 bp for the full-length Nluc-containing fragment and 361 bp for the WT fragment.

Figure S2. The percentage of haplotypes detected in the Nluc region of each GC-modified Nluc recombinant virus. (A) The proportion of each haplotype at P10 in the GC-modified Nluc recombinant SARS-CoV-2 variants. (**B**) The proportion of each haplotype at P10 in the GC-modified Nluc recombinant HCV variants. (**C**) The proportion of each haplotype at P10 in the GC-modified Nluc recombinant JEV variants.

Figure S3. Supplementary characterization of the additional Nluc-mL variant. (A) The luciferase activity of wildtype and the three versions of GC-modified Nluc under the HSV-TK promoter were evaluated after transfection into HEK293T cells. The bar graphs show the normalized data (Nluc/firefly luciferase activities). (n = 3) (**B**) RNA secondary-structure predictions for Nluc-mL.

Figure S4. Gel electrophoresis of the recombinant HCVs after ten rounds of serial passage. Gel electrophoresis of PCR amplicons of the Nluc region purified from the

recombinant HCVs after ten rounds of passaging. M indicates DNA marker as reference. The expected PCR product sizes were 1,070 bp for the full-length Nluc-containing fragment and 554 bp for the WT fragment.

Kawahara et al., Supplementary Fig. 1

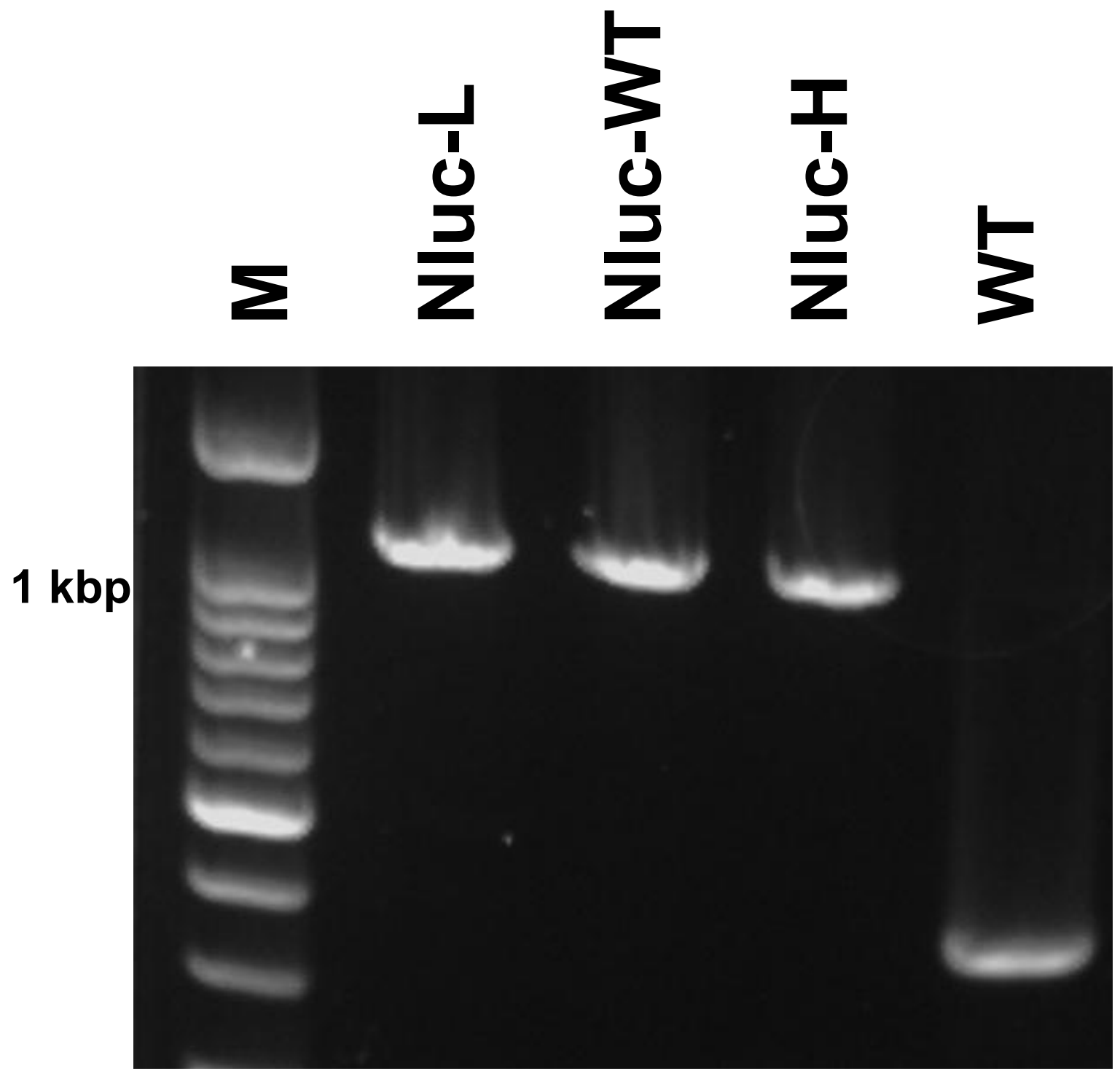

Kawahara et al., Supplementary Fig. 2

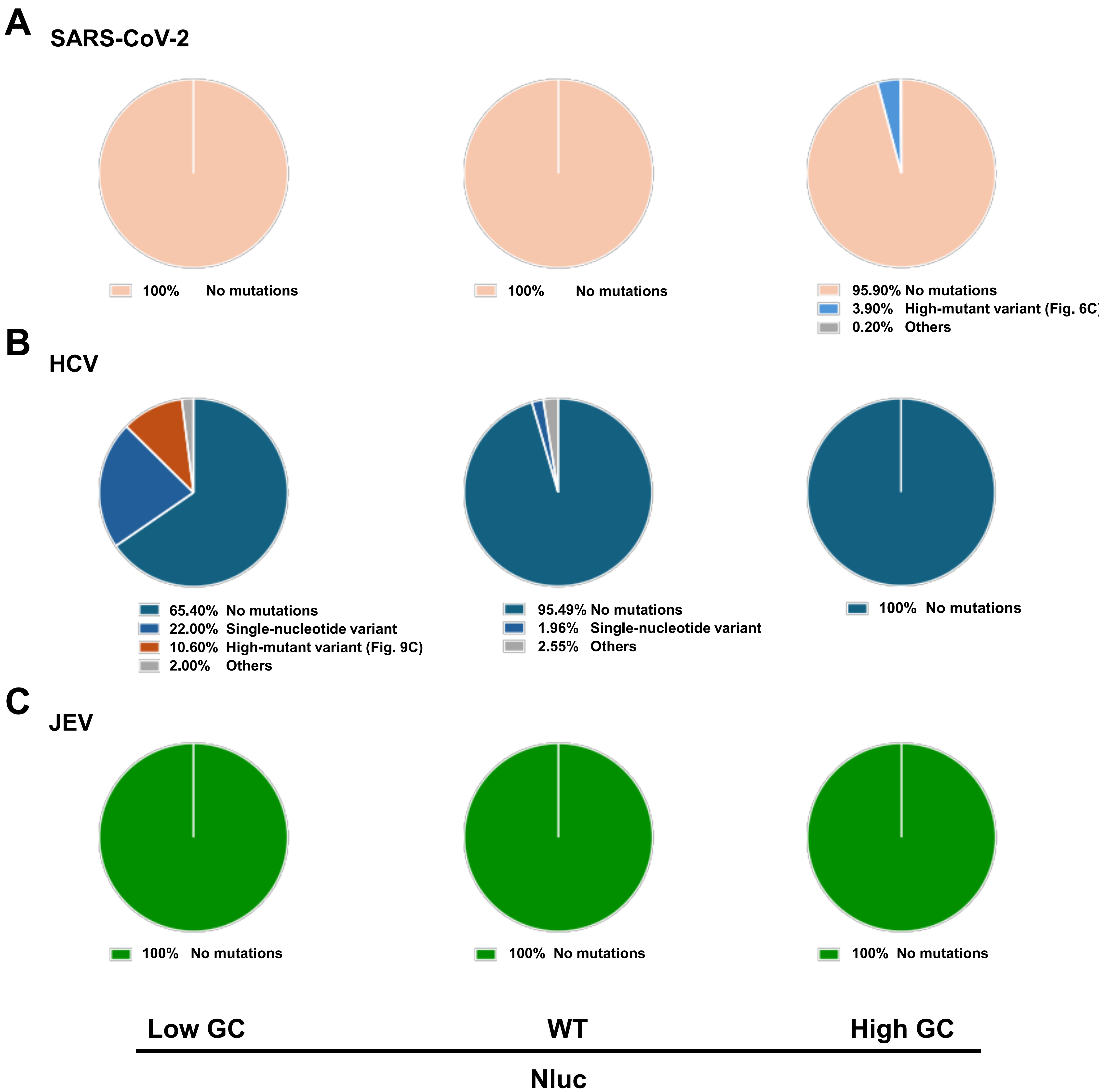

Kawahara et al., Supplementary Fig. 3

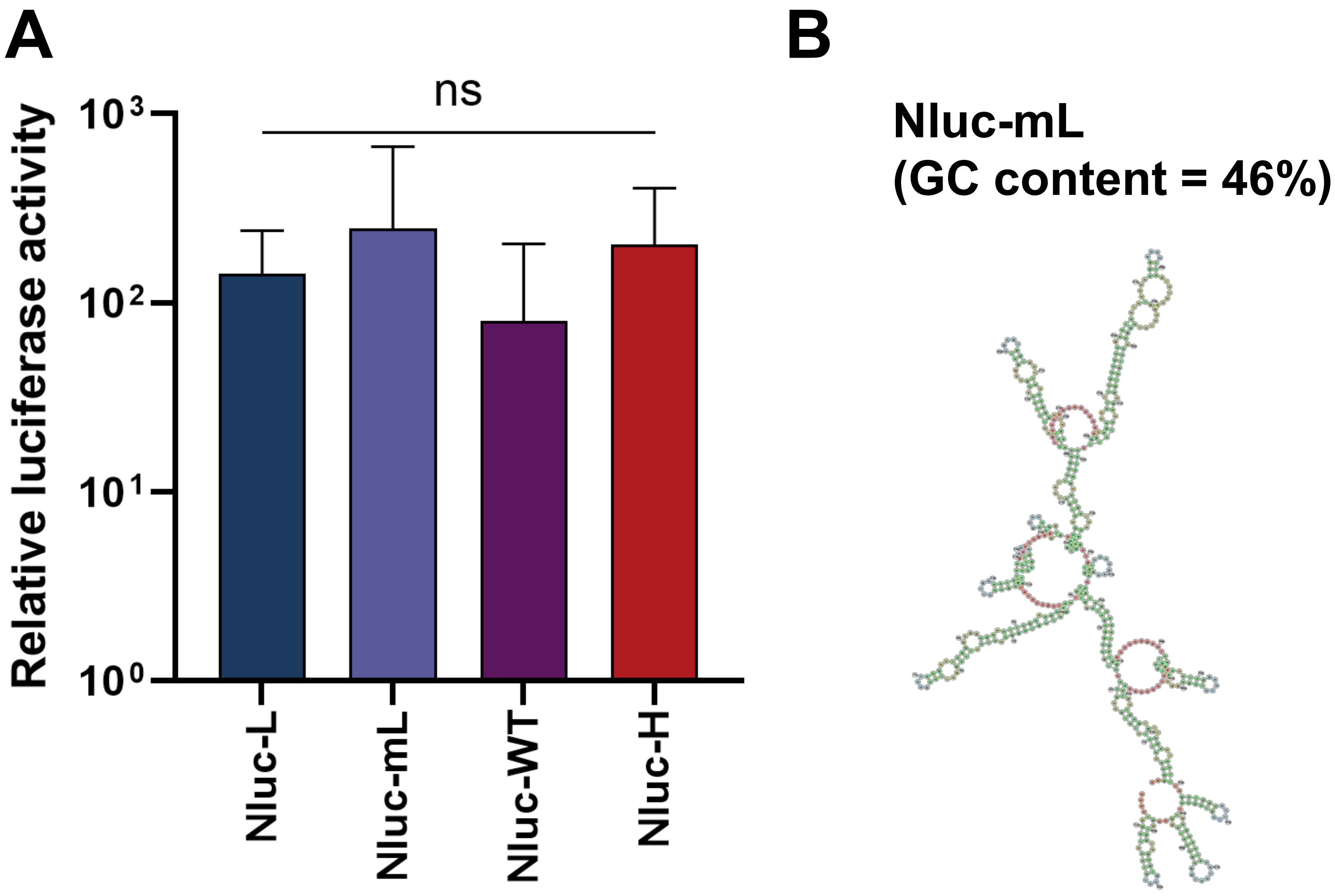

Kawahara et al., Supplementary Fig. 4

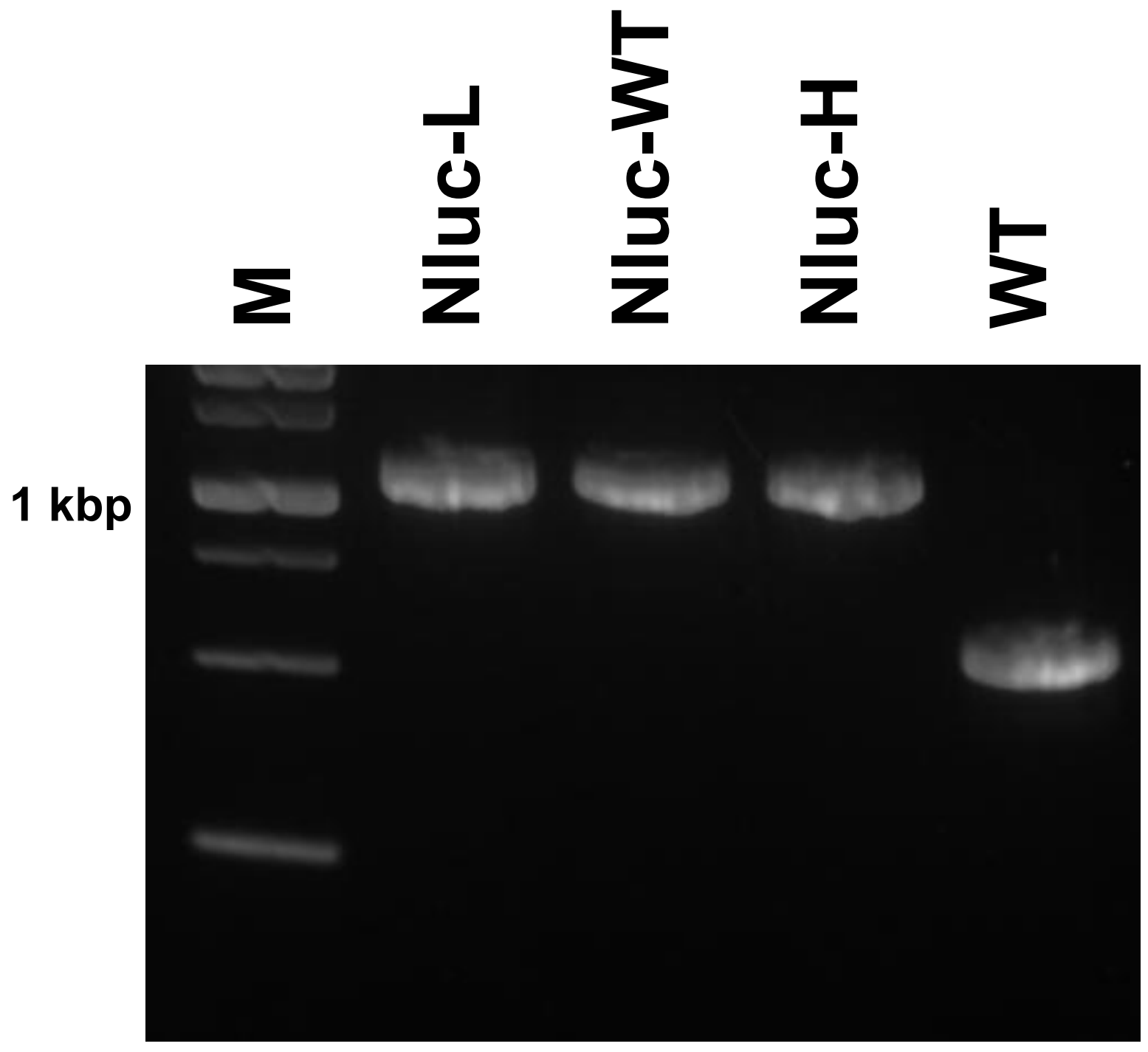

Supplement: Supplementary file 1 [file jvi.00882-26-s0001.pdf]
